# The Effects of Marital Status, Fertility, and Bereavement on Adult Mortality in Polygamous and Monogamous Households: Evidence From the Utah Population Database

**DOI:** 10.1007/s13524-020-00918-z

**Published:** 2020-09-15

**Authors:** Kieron J. Barclay, Robyn Donrovich Thorén, Heidi A. Hanson, Ken R. Smith

**Affiliations:** 1 Max Planck Institute for Demographic Research, Rostock, Germany; 2 Department of Sociology, Stockholm University, Stockholm, Sweden; 3 Swedish Collegium for Advanced Study, Uppsala, Sweden; 4 Centre for Sociological Research, Katholieke Universiteit Leuven, Leuven, Belgium; 5 Population Science, Huntsman Cancer Institute, University of Utah, Salt Lake City, UT USA; 6 Department of Surgery, University of Utah, Salt Lake City, UT USA; 7 Department of Family and Consumer Studies, University of Utah, Salt Lake City, UT USA

**Keywords:** Polygamy, Mortality, Marital status, Fertility, Bereavement

## Abstract

Although the associations among marital status, fertility, bereavement, and adult mortality have been widely studied, much less is known about these associations in polygamous households, which remain prevalent across much of the world. We use data from the Utah Population Database on 110,890 women and 106,979 men born up to 1900, with mortality follow-up into the twentieth century. We examine how the number of wife deaths affects male mortality in polygamous marriages, how sister wife deaths affect female mortality in polygamous marriages relative to the death of a husband, and how marriage order affects the mortality of women in polygamous marriages. We also examine how the number of children ever born and child deaths affect the mortality of men and women as well as variation across monogamous and polygamous unions. Our analyses of women show that the death of a husband and the death of a sister wife have similar effects on mortality. Marriage order does not play a role in the mortality of women in polygamous marriages. For men, the death of one wife in a polygamous marriage increases mortality to a lesser extent than it does for men in monogamous marriages. For polygamous men, losing additional wives has a dose-response effect. Both child deaths and lower fertility are associated with higher mortality. We consistently find that the presence of other kin in the household—whether a second wife, a sister wife, or children—mitigates the negative effects of bereavement.

**Electronic supplementary material:**

The online version of this article (10.1007/s13524-020-00918-z) contains supplementary material, which is available to authorized users.

## Introduction

The detrimental effect of losing a spouse on the mortality of the surviving partner is one of the most consistently observed patterns in the social sciences (Elwert and Christakis 2008a; Hu and Goldman 1990; Martikainen and Valkonen 1996; Mineau et al. 2002; Smith and Zick 1996). Although practically all individuals will experience some form of bereavement over the life course, the loss of a spouse is a particularly stressful event and can induce depression; feelings of anxiety, guilt, and hopelessness (Stroebe et al. 2007); and related behavioral changes. A meta-analysis has shown that the widowhood effect is typically worse for men than for women: mortality increases by about 27% for men following the death of a spouse, compared with about 15% for women (Shor et al. 2012). These main effects, however, suppress substantial effect heterogeneity. Studies have shown that the widowhood effect is moderated by a variety of factors, including age, time since widowhood, geographical region, historical period, and race (Elwert and Christakis 2006; Shor et al. 2012; Smith and Zick 1996). To date, however, research has focused on the widowhood effect in monogamous marriages, failing to consider whether the widowhood effect on mortality might vary between monogamous and polygamous marriages. *Polygamy* refers to having multiple spouses simultaneously, and *polygyny* refers specifically to marriages with one man and multiple women. Although monogamous marriages are today the most common type of marriages, more than 80% of preindustrial societies permitted polygamy, most commonly in the form of polygyny (Gray 1998; Murdock 1967; Murdock and White 1969). Furthermore, polygyny remains legal or accepted across much of Africa and the Middle East today (Al-Krenawi 2014; Bledsoe 1990; Jacoby 1995; Smith-Greenaway and Trinitapoli 2014).

In this study, we extend research on the widowhood mortality effect by examining whether the effect of losing a spouse differs between monogamous and polygamous marriages as well as the relative effects of different forms of family bereavement on subsequent mortality. For both men and women, we compare the relative effect of partner loss to the effect of losing a child and explore whether child deaths exacerbate the negative effects of partner loss. By studying the relative effects of different types of bereavement, we hope to shed light on the different mechanisms by which the widowhood mortality effect has been hypothesized to operate. To address these questions, we use the Utah Population Database (UPDB), a remarkable data source with individual-level information and kinship linkages from the eighteenth century to the present day.

### Marital Status and Health

Research has consistently demonstrated that marital status is an important social determinant of health and mortality. Studies have shown that those who are married have better physical and mental health as well as lower mortality (Drefahl 2012; Hu and Goldman 1990; Manzoli et al. 2007). Positive selection into marriage explains part of this advantage (Mastekaasa 1992; Murray 2000), but spouses also provide social, emotional, and financial support, monitor one another’s health and health behaviors, and provide connections to a larger social network. Research has suggested that marriage benefits men more than women, and married men eat more healthily and drink less alcohol than men who are not married (Umberson 1987; Umberson et al. 1992). Some studies suggest that the protective effect of marriage for women is derived primarily from the financial benefits of marriage (Lillard and Waite 1995). Although women do also derive health benefits from marriage, they often have more dependable sources of social support outside of marriage than men and are therefore less reliant on their partner (Antonucci and Akiyama 1987).

The protective elements of marriage—social support, normative expectations around health and health behaviors, and financial resources—are typically lost upon the death of a partner, and the death of a spouse often induces acute emotional distress for the survivor (Stroebe et al. 2007). Widowed men and women tend to adopt less healthy behaviors, particularly in the period directly after bereavement, but this reaction is more pronounced for men (Jin and Chrisatakis 2009; Umberson 1992). The negative effect of widowhood is worse at younger ages for both men and women, but the gender differences in the widowhood effect on mortality narrow with increasing age (Shor et al. 2012). A considerable part of the widowhood mortality effect seems to be attributable to reliance on the partner, and some research suggests that individuals in marriages with a less pronounced division of labor are less negatively affected by widowhood because they are more self-sufficient (Elwert and Christakis 2006).

Although spousal loss clearly has a negative effect on the surviving partner, some research suggests that the benefits of marriage can persist even after partner death because the preceding years have shaped patterns of health behaviors that are not immediately unlearned (Elwert and Christakis 2006). Greater health and better habits are also likely to increase the probability that an individual might remarry following widowhood (Mineau et al. 2002; Smith et al. 1991). Although widowed individuals almost always have worse health than married individuals, those who are widowed may maintain better health than those who were never married, even after selection processes are taken into account.

#### Potential Sources of Bias

Given that many careful studies have found a widowhood mortality effect on the mortality of the surviving spouse, the overall consensus is that there is a causal effect of widowhood on mortality and that the observed pattern is not due to statistical confounding (Shor et al. 2012). Nevertheless, a widowhood mortality effect could be produced by bias from homophily and shared environmental exposures (Smith and Zick 1994).

*Homophily* refers to the empirical regularity whereby similar people have higher rates of contact and are more likely to group together than dissimilar people (McPherson et al. 2001). A wealth of studies has demonstrated patterns of positive assortativity in the formation of social relationships (e.g., McPherson et al. 2001). Studies have indicated that partners in romantic relationships tend to be similar on a variety of different factors, including educational levels, health, and health behaviors. If spouses select into a relationship based on similar health or health behaviors, a correlation in the timing of the death of a husband and wife could be attributable to sharing similar characteristics and health behaviors rather than any direct effect of partner loss (Goldman 1993; Smith and Zick 1994), although research that has attempted to tackle this issue directly suggests that homophily bias is not the key driver of the observed widowhood mortality effect (Elwert and Christakis 2008b).

Sharing the same household environment, and therefore common risk factors for mortality, could also induce a correlation in the timing of death between a wife and a husband (Smith and McClean 1998; Smith and Zick 1994). Such shared risk factors might include arsenic in the groundwater (Lewis et al. 1999), which is a common problem in the southwestern United States (Welch et al. 1988). Likewise, members of households engaged in farming would all suffer from crop failure or severe weather conditions affecting agricultural production. Children in the household are also a shared exposure to both mother and father; children affect resource distribution, and adverse events, such as a child death, would affect both parents as well. No study to date has been able to distinguish the relative importance of a genuine bereavement effect from a shared environment effect in producing the widowhood mortality effect.

### The Effect of Widowhood on Mortality in Polygamous Marriages

The benefits of marriage may have been particularly pronounced in the nineteenth century in the territory that would later become the U.S. state of Utah.^1^ This region in western North America beheld a frontier society where settlers had to contend with a harsh physical environment (Bean et al. 1990), limited medical knowledge and facilities (Mineau et al. 2002), and conflict with displaced Native Americans (Anderson 1942). Food supplies were also unreliable because of the risk of crop failure and the relative geographic isolation of many households (Arrington and May 1975; Hanson and Smith 2013).

Men who could afford to marry multiple women in this context typically had greater financial sources, high status in the Church of Jesus Christ of Latter-day Saints, or both (Ulrich 2017; Young 1970). These financial and social resources are likely to have mitigated risks in times of scarcity (Lawson et al. 2015). Men and women in plural marriages would also be expected to have lower mortality by virtue of their church affiliation, from the community support available from fellow members as well as the associated tenets that forbade the consumption of alcohol or tobacco (Bush 1993). On the basis of differences in status and health behaviors, we would expect that men in polygamous marriages in Utah would have lower mortality than men in monogamous marriages. Furthermore, in plural marriages, if one wife died, the surviving husband would still have access to emotional support and help raising any children from his surviving wives. The presence of multiple adult members in the marriage could function as insurance against misfortune and offer the whole family greater resiliency. Previous studies have shown that the presence of other kin is an important factor for mitigating the negative consequences of widowhood (Mineau et al. 2002; O’Bryant 1988), and an additional surviving spouse would likely play a similar moderating role. However, if the widowhood mortality effect is attributable to shared environmental factors, the death of any household member—whether a wife or a child—is likely to increase mortality in a similar way for the surviving husband.

We also expect that women in plural marriages would have lower mortality than women in monogamous marriages. Some have argued that at the aggregate level, a polygynous marriage system offers benefits to women because they should have greater bargaining power and be able to marry “higher-quality” men (Becker 1991: chapter 3). This theoretical perspective has also found some empirical support in contemporary sub-Saharan Africa (see, e.g., Lawson et al. 2015). At the individual level, women in plural marriages in nineteenth-century Utah are certainly likely to have benefited from having a husband with greater resources and higher social status. Furthermore, in the event of bereavement, women in plural marriages who lost either a husband or sister wife^2^ would still have access to various forms of support from the surviving adult household members. We note that women could experience the negative effects of bereavement regardless of whether it was the husband or the sister wife who passed.

To the extent that previous research has shown that the health benefits of marriage in contemporary societies primarily extend from access to financial resources from the husband (Lillard and Waite 1995), the death of a husband in a plural marriage might be expected to have a greater impact than the death of a sister wife on a woman in a polygynous marriage. However, if a shared environmental exposure is responsible for producing the widowhood mortality effect, then we would expect that the death of a sister wife and the death of the husband would have a similar effect on the mortality of the surviving wife. If the death of the husband has a significantly greater effect on the mortality of a surviving wife than the death of a sister wife, then that would provide persuasive evidence for a direct effect of spousal loss on subsequent mortality over a spurious pattern that is a product of a shared environment.

The relative effect of the death of a husband versus a sister wife may have varied by the economic activity of the household. Households in this context tended to observe a traditional gender division of labor, with husbands acting as breadwinners and wives acting as homemakers (Ulrich 2017; Young 1970). In most households, this would leave women more vulnerable in the event of the death of the husband. However, for farming households, the loss of any adult household member or teenage children may have had serious consequences for farm productivity. U.S. Census data show that 60% of households in Utah were classified as farming households in 1850 and 1860, falling to 43% in 1870 and to approximately 35% in both 1880 and 1900 (Ruggles et al. 2019). The loss of a husband or sister wife might have a similarly negative effect on the surviving household members among households dependent on farming. The death of a husband may have meant a significant or complete loss of household income for the surviving wives and children in nonfarming households. The death of any adult household members would have affected productivity—and therefore household income—in farming households. To assess this empirically, we examine whether the widowhood effect on mortality in monogamous and polygamous households varied by whether the husband’s occupation was recorded as a farmer.

Marriage order may also have played an important role in the survival of women in polygamous marriages (Bove and Valeggia 2009; Jankowiak et al. 2005). Most of the empirical literature examining the relationship between marriage order and wife outcomes is based on studies of contemporary polygynous households in Africa, but there are also some studies based on historical households with members of the Church of Jesus Christ of Latter-day Saints. Studies have generally indicated that first and earlier-ranked wives have higher fertility than later wives (Bean and Mineau 1986; Gibson and Mace 2007) and that the children of first wives have better nutrition (Gibson and Mace 2007) and lower mortality (Strassmann 1997). First wives also spend time with the husband in a monogamous marriage before later wives join (Josephson 2002). To the extent that exclusive access to resources from the husband affects health, this would benefit the health of the first wife and her children. Furthermore, a longer tenure within the household may give the first wife greater status and control over the allocation of household resources (Mulder 1989). During some periods in nineteenth-century Utah, being the first wife would also have granted a favorable position in terms of inheritance following the death of the husband (Young 1970: chapter 13). In nineteenth-century Utah, second and later wives also tended to be older than the first wife was at the time of marriage (Josephson 2002), although they were almost always younger than the first wife at the time that they joined the marriage. This research suggests that after taking age into account, higher marriage order may be associated with a higher risk of mortality. However, it may be that later wives attracted the particular favor of the husband, which may have led to relative survival advantages within the family.

Children may also play an important role in moderating the effects of marital status on mortality. Fertility was high in nineteenth-century Utah, with total fertility rates above 10 across the nineteenth century (Skolnick et al. 1978). Although child mortality was relatively low in Utah compared with the rest of the United States in the nineteenth century, rates were much higher than they are today (Lynch et al. 1985). The death of a child has its own independent, and potentially emotionally devastating, negative effect on the parents: research has shown that child deaths are associated with higher levels of parental, and particularly maternal, mortality (Rostila et al. 2012). Child deaths may also be indicative of harmful shared environmental exposures. In a study using historical data from Sweden, Belgium, and the Netherlands, Alter et al. (2007) found that the death of a husband was particularly detrimental when a woman had multiple children and lost her husband at a young age, implying that the burden of supporting a large household alone was more likely to lead to an early grave. However, having surviving children may also provide emotional support and also renewed motivation and purpose in life following partner loss.

Children older than age 6 would also contribute to the household economy, with increasing responsibilities as they grew older (Young, 1970:244). We examine how the death of a child affects mortality relative to the death of adulthood household members, as well as how child deaths and the number of children ever born statistically interact with partner loss.

## Data and Methods

### Data

This study is based on data from the Utah Population Database (UPDB). The central component of UPDB is an extensive set of Utah family histories, in which family members are linked to demographic and medical information. The UPDB contains information on more than 11 million individuals, including the genealogies of the founders of Utah and their descendants, sourced from the Genealogical Society of Utah. The genealogy records for early migrants, their families, and their descendants represent birth cohorts that date back to near 1760. These early records provide basic demographic information on almost 200,000 families (more than 1.6 million individuals). These early records have been linked across generations, and in some instances, the records encompass as many as 11 generations. Individuals in these data may or may not have an affiliation to the Church of Jesus Christ of Latter-day Saints and may have lived in other states or countries. The genealogy records have been linked to other data sets, including Utah birth and death certificates, cancer records, the Social Security Death Index, and U.S. Censuses from 1880 to 1940. Multiple sources of death information are available in UPDB for parents and their children, including genealogy records, Utah death certificates beginning in 1904, and the Social Security Death Index. The analysis for this study is limited to individuals born up to 1900. We follow individuals from the point at which they marry until the time of death.

Data from the UPDB indicate that the frequency of polygamous men as a fraction of all married men was highest among men born in 1833, at 17.8% (Moorad et al. 2011; Smith and Kunz 1976). Decennial censuses showed that the proportion of the population in polygamous families reach a peak around 1860, when 43.6% men, women, and children were in a polygamous family of some sort, although this proportion was heavily dominated by children (Bushman 2008). After this peak, the proportion declined over time, until the equivalent figure was 25% in 1880 and 7.1% in 1900 (Bushman 2008).

### Marital Status

Our key explanatory variable is marital status, which we treat as a time-varying covariate. This is important because we distinguish between monogamous and polygamous marriages in our analyses, and a monogamous marriage does not become polygamous until at least a second woman joins that marriage. Furthermore, it is critical to model widowhood, the death of more than one wife, the death of a sister wife, and remarriage after widowhood using a time-varying approach. As a result, the relative risks estimated in our event history analyses reflect the hazard of mortality of an individual in a given marital state, and the same individual may contribute exposure to multiple different categories of our marital status variables (e.g. married, widowed, remarried). We conduct separate analyses for men and women. For men, we top-code the number of wives at 4+ (few men had more than four wives). The sample size for the analytical population is 110,890 for women and 106,979 for men.

Our analyses for women include women who had up to three husbands, which is explained by remarriage following the death of the partner, but only a tiny fraction of women had more than two husbands because that would require the death of two husbands as well as a second remarriage. As a result, we collapse first and second monogamous remarriages, and second and third husband deaths in monogamous marriages, into the same categories. We model not only the death of husbands but also the deaths of sister wives. We use 20 categories (states) for marital status for our analyses, and these categories should be read from the perspective of the index person in the marriage:

1. First monogamous marriage
2. Widowed in first monogamous marriage
3. Remarried (second or third monogamous marriage)
4. Widowed again (second or third monogamous marriage)
5. Polygamous marriage with two wives where the husband and sister wife are both alive
6. Polygamous marriage with two wives where the sister wife has died, and the husband is alive
7. Polygamous marriage with two wives where the sister wife is alive, and the husband is dead
8. Polygamous marriage with two wives where the husband and sister wife are both dead
9. Polygamous marriage with three wives where the husband and sister wives are all alive
10. Polygamous marriage with three wives where one sister wife has died, one sister wife is alive, and the husband is alive
11. Polygamous marriage with three wives where both sister wives have died, and the husband is alive
12. Polygamous marriage with three wives where both sister wives are alive, and the husband is dead
13. Polygamous marriage with three wives where one sister wife is dead, one sister wife is alive, and the husband is dead
14. Polygamous marriage with three wives where both sister wives are dead, and the husband is dead
15. Polygamous marriage with four or more wives where the husband and sister wives are all alive
16. Polygamous marriage with four or more wives where some of (not all) the sister wives are dead, and the husband is alive
17. Polygamous marriage with four or more wives where all the sister wives are dead, and the husband is alive
18. Polygamous marriage with four or more wives where all the sister wives are alive, and the husband is dead
19. Polygamous marriage with four or more wives where some of (not all) the sister wives are dead, and the husband is dead
20. Polygamous marriage with four or more wives where all the sister wives are dead, and the husband is dead

Because very few women have more than one polygamous husband, which would require being widowed in the first polygamous marriage and then remarrying into another polygamous marriage, we collapse all higher-order polygamous marriages into the same categories. For example, if a women joins a polygamous marriage, and is then widowed, and then remarried into a second polygamous marriage, she could move from state 5 to state 7, and back to state 5 again in the second marriage. We have also conducted analyses where we model second and third marriages as completely separate states, but the patterns are qualitatively similar across first, second, and third marriages, and so we collapse the categories to increase precision.

For our interaction analyses, we also run models with a simplified set of categories for polygamous marriages to reduce some of the inherent complexity, collapsing polygamous marriages into those where (1) all sister wives and the husband are alive; (2) some, but not all, of the marriage group are deceased; and, (3) all members of the marriage group, except for the index person, are deceased. Among women, we also study the relationship between marriage order and mortality.

Our analyses for men include a marital status variable with 18 categories:

1. First monogamous marriage
2. Widowed from first monogamous marriage
3. First monogamous remarriage
4. Widowed from second monogamous marriage
5. Second monogamous remarriage
6. Widowed from second monogamous remarriage
7. Polygamous marriage with two wives, where both wives are alive
8. Polygamous marriage with two wives, where one wife has died
9. Polygamous marriage with two wives, where both wives are deceased
10. Polygamous marriage with three wives, where all wives are alive
11. Polygamous marriage with three wives, where one wife has died
12. Polygamous marriage with three wives, where two wives have died
13. Polygamous marriage with three wives, where all three wives are deceased
14. Polygamous marriage with four wives, where all wives are alive
15. Polygamous marriage with four wives, where one wife has died
16. Polygamous marriage with four wives, where two wives have died
17. Polygamous marriage with four wives, where three wives have died
18. Polygamous marriage with four wives, where all four wives are deceased

As mentioned earlier, an index individual may move between these different states, and may contribute exposure to each state that he occupies in the event history analysis. For example, a man in a monogamous marriage who then takes a second wife will move from state 1 to state 7; and if he then takes on a third wife, and none of the wives have died, he will move to state 10. If he outlives all three of his wives, he would then move from state 10 through states 11, 12, and 13.

For our interaction analyses, we also run models with a simplified set of categories for polygamous marriages. We collapse polygamous marriages into those where (1) all sister wives are alive; (2) some of, but not all, the sister wives have died; and (3) all the sister wives have died.

### Statistical Analyses

To study the relationship between marital status and mortality, we use survival analysis in the form of Cox proportional hazard models (Cox 1972). The general proportional hazards model is expressed as:

$$ h\left(\left.t\right|{X}_1,\dots, {X}_k\right)={h}_0(t)\exp \left({\sum}_{j=1}^k{\upbeta}_j{X}_j(t)\right), $$

where *h*(*t|X*_1_, . . . , *X*_*k*_) is the hazard rate for individuals with characteristics *X*_1_, . . . , *X*_*k*_ at time *t*; *h*_0_(*t*) is the baseline hazard at time *t*; and β_*j*_, *j* = 1, . . . , *k* are the estimated coefficients. The failure event in our analysis is the death of the index person, and the baseline hazard in our model *h*_0_(*t*) is time since first marriage. We censor on the time when the person is lost to follow-up or outmigration from Utah, which is recorded in the UPDB.

We also estimate a series of stratified Cox models, which allows us to stratify the baseline hazard by different groups, based on the assumption that there are unobserved factors particular to each category that may lower or elevate the shared hazard. This allows us to effectively adjust for factors that are shared within strata to the extent that they are time-invariant (Allison 2009). The stratified Cox model takes the following form, where the hazard for an individual from stratum *s* is

$$ {h}_s\left(\left.t\right|{X}_1,\dots, {X}_k\right)={h}_{0s}(t)\exp \left({\sum}_{j=1}^k{\upbeta}_j{X}_j(t)\right), $$

where *h*_0*s*_(*t*) is the baseline hazard for stratum *s*, *s* = 1, . . . , *S*. Our general strategy is to stratify by age at first marriage in single-year age groups because the baseline hazard in our models is time since first marriage. This effectively allows us to adjust for age differences in mortality. We also employ stratified Cox models to examine the effect of marriage order on the mortality of women in polygamous marriages; in these models, we stratify by the shared husband ID. We estimate the following models for women:

1 $$ \log \kern0.3em h(t)={\upbeta}_1{Marital}_{ij}+{\upbeta}_2{A}_{ij}+{\upalpha}_j $$

2 $$ \log \kern0.5em h(t)={\upbeta}_1{Marital}_{ij}+{\upbeta}_2{A}_{ij}+{\upbeta}_3{B}_{ij}+{\upalpha}_j, $$

where log *h*_*i*_(*t*) is the log hazard of mortality, α_*j*_ is the shared group by age at first marriage *j*, and the index *ij* refers to the individual *i* in that group *j*; *Marital*_*ij*_ is entered into the model as a series of 20 dummy variables based on the categories of marital status for women described in detail in the preceding section; and *A*_*ij*_ is a set of control variables including birth cohort and church affiliation. Birth cohort is a categorical variable that groups together those who are born in the 1700s, who are few, and 10-year groupings for those born from 1800 to 1900. We adjust for affiliation with the Church of Jesus Christ of Latter-day Saints because it is linked directly to the likelihood of entering a plural marriage in the first place and is also related to mortality risk. Adherents have a proscription from alcohol and tobacco use; the emphasis placed on community and social integration, as well as monthly fasting, means that health outcomes for both the men and women in our sample are related to affiliation. This variable has three categories: (1) no affiliation, (2) inactive, and (3) active. Those who are active pledged to abide by the doctrine of the religion. Inactive members are those who were baptized, but we have no evidence that they later pledged to abide by the doctrine of the religion.

In Model 2, we introduce an additional set of control variables, *B*_*ij*_, for whether the woman was born in Utah, the occupational status of the husband, whether the husband was a farmer, age difference from the husband, biological parity, and child deaths. Our measure for the socioeconomic status (SES) of the husband is a NPSES (Nam-Powers-Boyd) occupational status score for a measure of occupational status, where scores range from 0 to 99 (Nam and Boyd 2004). We include this variable in the models split into 11 categories (1–9, 10–19, . . . , 90–99, and missing). The variable for whether the husband was a farmer is entered as a separate dummy variable. We control for the SES of the husband in both our analyses of men and of women because household SES was best determined by the husband’s occupational status during this period. Age difference between the husband and wife is operationalized as husband age minus wife age and is split into seven categories (<–9; –9 to –5; –4 to –1; –1 to 1; 1 to 4; 5 to 9; >9). We control for age difference between the husband and wife because research suggests that the age difference between partners affects their hazard of mortality (Drefahl 2010). If a woman is widowed, she retains the values from the deceased husband for the variables husband occupation, whether the husband was a farmer, and age difference from the husband until the time at which she might remarry, at which point those variables are updated to reflect the characteristics of the new husband. The variables for parity (0, 1, . . . , 9, 10+) and child deaths (0, 1, 2, 3, 4, 5+) are time-varying covariates, updating with each subsequent birth and death. We also repeat Models 1 and 2 using a simplified version of marital status, with only seven categories, detailed earlier.

We also estimate several models examining the statistical interaction between the simplified version of our marital status variable for women (seven categories) and several key variables:

3 $$ \log \kern0.3em h(t)={\upbeta}_1{Marital}_{ij}\times {ChildDeaths}_{ij}+{\upbeta}_2{A}_{ij}+{\upbeta}_3{B}_{ij}+{\upalpha}_j $$

4 $$ \log\;h(t)={\upbeta}_1{Marital}_{ij}\times {Parity}_{ij}+{\upbeta}_2{A}_{ij}+{\upbeta}_3{B}_{ij}+{\upalpha}_j $$

5 $$ \log \kern0.3em h(t)={\upbeta}_1{Marital}_{ij}\times {HusbandFarmer}_{ij}+{\upbeta}_2{A}_{ij}+{\upbeta}_3{B}_{ij}+{\upalpha}_j, $$

where *ChildDeaths*_*ij*_ is a time-varying covariate for experience of child deaths (0, 1, 2+), *Parity*_*ij*_ is a time-varying covariate for number of children ever born (0, 1–3, 4–6, 7–9, 10+), and *HusbandFarmer*_*ij*_ is a binary variable for whether the husband was a farmer. In Models 3–5, control variable set B omits the variable directly associated with the interaction variable of interest.

Finally for women, we also estimate models to examine how marriage order affects the mortality of women in first marriages that were polygamous:

6 $$ {\displaystyle \begin{array}{l}\log\;h(t)={\upbeta}_1{Order}_{ij}+{\upbeta}_2{BirthYear}_{ij}+{\upbeta}_3{HusbandDead}_{ij}+{\upbeta}_4{HusbandOcc}_{ij}\kern0.3em +\\ {}{\upbeta}_5{HusbandFarmer}_{ij}+{\upalpha}_j\end{array}} $$

7 $$ \log \kern0.3em h(t)={\upbeta}_1{Order}_{ik}+{\upbeta}_2{BirthYear}_{ik}+{\upbeta}_3{HusbandDead}_{ik}+{\upbeta}_4{AFM}_{ik}+{\upzeta}_k, $$

where *Order* refers to marriage order (1, 2, 3, 4, 5+), *BirthYear* is a continuous variable for birth year, *HusbandDead* is a time-varying covariate for whether the husband is alive or deceased, *HusbandOcc* is husband occupational status, and *HusbandFarmer* is whether the husband is a farmer. Model 6 stratifies by age at first marriage, and Model 7 stratifies by shared husband ID, ζ_*k*_. Nested within each husband, the wives share the same baseline hazard, which adjusts for factors that are shared by wives to the extent that they are time-invariant (Allison 2009). In Model 7, we also estimate cluster-adjusted robust standard errors clustered at the level of the shared husband ID (Lin and Wei 1989), and we explicitly control for age at first marriage (16*,* 17, . . . , 59*,* 60+), *AFM*_*ik*_. We conduct separate analyses by the number of sister wives (2, . . . , 5+), as well as a pooled analysis. In these analyses, we condition on all preceding sister wives being alive at the time that the index person joins the marriage. We introduce this condition because higher-order wives might be replacements for previously deceased wives in polygamous marriages, and this produces a statistical artifact whereby earlier wives have a relative mortality risk much greater than higher-order wives.

In our analyses of men, we estimate the following models:

8 $$ \log \kern0.3em h(t)={\upbeta}_1{Marital}_{ij}+{\upbeta}_2{A}_{ij}+{\upalpha}_j $$

9 $$ \log \kern0.3em h(t)={\upbeta}_1{Marital}_{ij}+{\upbeta}_2{A}_{ij}+{\upbeta}_3{C}_{ij}+{\upalpha}_j, $$

where *Marital*_*ij*_ is entered into the model as a series of 18 dummy variables based on the categories of marital status for men described in detail in the preceding section; control set *A*_*ij*_ refers to birth cohort and church affiliation, and control set *C*_*ij*_ includes variables for whether the man was born outside Utah, the occupational status of the man, whether the man was a farmer, biological parity (0, 1, . . . , 10*,* 11–14*,* 15–19*,* 20+), and child deaths (0, 1, 2, 3, 4, 5+). The variables for parity and child deaths are time-varying covariates, updating with each subsequent birth and death. Polygamous men whose wives die generally will have many children, and rearing these children would be a stressor. We do not control for age difference in our analyses of men because there is no single value for age difference between husband and wife for men who have multiple wives. We prefer to omit the covariate for spousal age difference rather than to assume the mean age difference across multiple wives.

We also estimate models examining the statistical interaction between the simplified version of our marital status variable for men (seven categories) and key variables:

10 $$ \log \kern0.3em h(t)={\upbeta}_1{Marital}_{ij}\times {ChildDeaths}_{ij}+{\upbeta}_2{A}_{ij}+{\upbeta}_3{C}_{ij}+{\upalpha}_j $$

11 $$ \log\;h(t)={\upbeta}_1{Marital}_{ij}\times {Parity}_{ij}+{\upbeta}_2{A}_{ij}+{\upbeta}_3{C}_{ij}+{\upalpha}_j $$

12 $$ \log\;h(t)={\upbeta}_1{Marital}_{ij}\times {Farmer}_{ij}+{\upbeta}_2{A}_{ij}+{\upbeta}_3{C}_{ij}+{\upalpha}_j, $$

where *ChildDeaths*_*ij*_ is a time-varying covariate for experience of child deaths (0, 1, 2+), *Parity*_*ij*_ is a time-varying covariate for number of children ever born (0, 1–3, 4–6, 7–9, 10+), and *Farmer*_*ij*_ is a binary variable for whether the man was a farmer. For Models 10–12, control variable set C omits the variable directly associated with the interaction variable of interest.

## Results

### Descriptive Statistics

Table 1 shows descriptive statistics for marital status for the analytical sample that we use in our analyses. Tables containing full descriptive statistics, including information on all covariates, can be found in Tables S1 and S2 in the online appendix. The unconditional rates in Table 1 show that women in polygamous marriages with a total of two wives have the lowest mortality when neither the sister wife nor the husband is dead. Mortality rates are higher in these polygamous marriages with two wives when the husband has died and the sister wife is alive in comparison with when the sister wife has died and the husband is alive, but the highest rates are seen among women where both the husband and sister wife have died.

**Table 1:** Descriptive statistics on marital status for analytical population: Men and women living in Utah, 1800–1900

		Monogamous			Polygamous		
Marital Status		Person-Years (%)	Deaths	Rate (10^–2^)	Person-Years (%)	Deaths	Rate (10^–2^)
Female							
Monogamous, first marriage		68.56	39,674	1.02			
Monogamous, first widowhood		19.55	58,743	5.30			
Monogamous, remarried		1.36	931	1.21			
Monogamous, widowed again		0.64	2,252	6.24			
Polygamous, two wives, husband and sister wife alive					1.80	1,011	0.99
Polygamous, two wives, husband alive, sister wife dead					0.22	251	2.02
Polygamous, two wives, husband dead, sister wife alive					0.35	750	3.80
Polygamous, two wives, husband dead, sister wife dead					0.27	1,166	7.60
Polygamous, three wives, husband and sister wives alive					0.73	392	0.95
Polygamous, three wives, husband alive, one sister wife dead					0.17	138	1.39
Polygamous, three wives, husband alive, two sister wives dead					0.02	28	3.08
Polygamous, three wives, husband dead, sister wives alive					0.17	318	3.37
Polygamous, three wives, husband dead, one sister wife dead					0.17	546	5.73
Polygamous, three wives, husband dead, two sister wives dead					0.07	304	8.08
Polygamous, four or more wives, husband and sister wives alive					0.42	219	0.92
Polygamous, four or more wives, husband alive, some sister wives dead					0.21	184	1.55
Polygamous, four or more wives, husband alive, all sister wives dead					0.00	4	4.96
Polygamous, four or more wives, husband dead, all sister wives alive					0.09	128	2.40
Polygamous, four or more wives, husband dead, some sister wives dead					0.24	701	5.08
Polygamous, four or more wives, husband dead, all sister wives dead					0.02	104	12.05
Male							
Monogamous, first marriage		85.32	66,394	1.67			
Monogamous, first widowhood		8.21	27,219	7.14			
Monogamous, second marriage		4.14	5,531	2.88			
Monogamous, second widowhood		0.34	1,399	8.94			
Monogamous, third marriage		0.18	308	3.72			
Monogamous, third widowhood		0.02	98	9.50			
Polygamous, two wives, both alive					0.98	656	1.44
Polygamous, two wives, one dead					0.25	530	4.47
Polygamous, two wives, both dead					0.04	193	10.91
Polygamous, three wives, all alive					0.17	135	1.70
Polygamous, three wives, one dead					0.15	222	3.28
Polygamous, three wives, two dead					0.05	143	5.93
Polygamous, three wives, all dead					0.00	27	13.79
Polygamous, four or more wives, all alive					0.05	38	1.55
Polygamous, four or more wives, one dead					0.06	74	2.83
Polygamous, four or more wives, two dead					0.03	50	3.93
Polygamous, four or more wives, three dead					0.01	38	6.97
Polygamous, four or more wives, all dead					0.00	24	20.64

A similar pattern can be seen in polygamous marriages with three wives. Mortality is lowest when both sister wives and the husband are alive, is higher if one sister wife dies, and is even higher if the second sister wife dies. If both sister wives are alive but the husband is dead, the mortality rates are higher than if both sister wives are dead and the husband is alive. If the husband is dead and one sister wife dies, the mortality rates are higher still, and unconditional mortality rates in polygamous marriages with three wives are highest of all when the husband and both sister wives are dead. Polygamous marriages with four or more wives show a similar pattern: the mortality is higher when some or all sister wives have died, rates are higher if the husband has died than if a sister wife dies, and rates are highest of all when all the sister wives and the husband are dead.

Women in monogamous marriages whose husbands are alive have the lowest mortality, and mortality is much higher among women who have been widowed. Those who are remarried have an unconditional mortality rate higher than those who were never widowed but much lower than those who were widowed. Women in monogamous marriages who were widowed again in the second or third marriage have the highest unconditional mortality rate of all women in monogamous marriages.

Table 1 also shows the descriptive statistics for the analyses based on men. Men in polygamous marriages with two wives who are still alive have the lowest unconditional mortality rates. Among men in polygamous marriages, the unconditional mortality rate increases monotonically as the proportion of wives who died of the total number of wives increases. Men in polygamous marriages who have lost all their wives have higher unconditional mortality rates than men in monogamous marriages who have been widowed. Among men in monogamous marriages, being widowed is associated with a higher unconditional rate of mortality, and remarrying is associated with a rate of mortality higher than men who have not been widowed although lower than those who had not remarried.

### Multivariate Regressions: Women

#### Marital Status

**Fig. 1:**
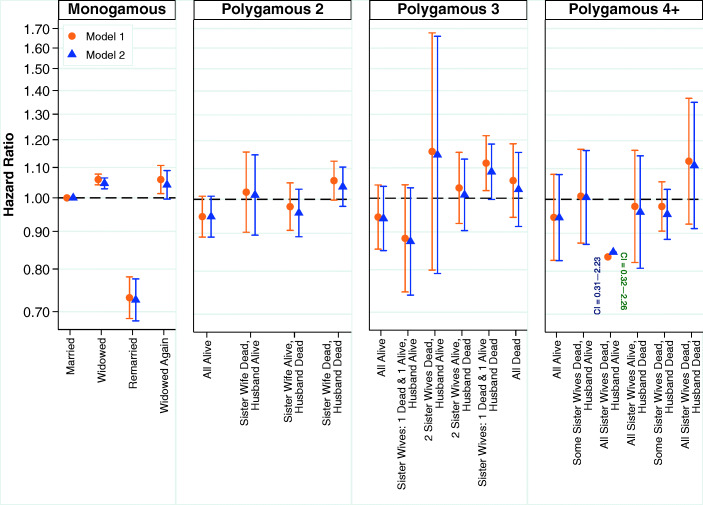
Multivariate estimates of mortality hazard for women using detailed marital status variable. Error bars are 95% confidence intervals. The estimates shown across the four panels are from the same model, and the common reference category is women in first monogamous marriages who have not been widowed. Estimates are based on Models 1 and 2.

The results from the multivariate analyses examining the relationship between marital status and mortality among women are shown in Fig. 1. The estimates shown across all four panels are from the same model, and the common reference category is women in monogamous relationships who have not been widowed; the split into separate panels is purely to assist interpretation of the results. A detailed table of results including covariates can be found in Table S3 in the online appendix. Compared with nonwidowed women in monogamous marriages, widowed women who had been living in a monogamous marriage have mortality that is 6% higher. Women in monogamous marriages who remarry have substantially lower mortality than women who were never widowed, with a hazard ratio of 0.73. Women who were widowed again following remarriage have a hazard of mortality very similar to women widowed from the first monogamous marriage. Remarkably, the results from Models 1 and 2 are very similar, indicating very little confounding or mediating from the socioeconomic characteristics of the husband, fertility behavior, or child deaths.

Women in polygamous marriages who have not experienced bereavement have lower mortality than women in monogamous marriages; and even when women in polygamous marriages experience the death of their husband and sister wives, their relative risk of mortality is not significantly greater than women in polygamous marriages who have not been widowed. Disregarding statistical significance, the point estimates suggest that women in polygamous marriages who experience the death of their husband as well as all sister wives have a similar hazard of mortality to women who are widowed in monogamous marriages.

**Fig. 2:**
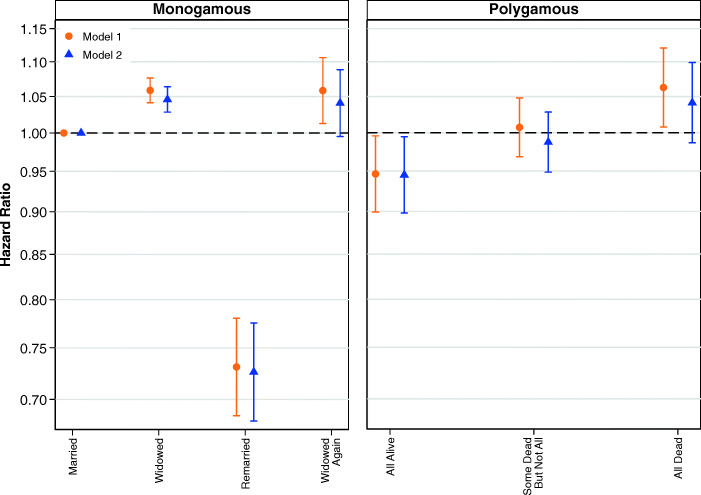
Multivariate estimates of mortality hazard for women using simplified marital status variable. Error bars are 95% confidence intervals. The estimates shown in the left and right panels are from the same model, and the common reference category is women in first monogamous marriages who have not been widowed. Estimates are based on Models 1 and 2.

We examine this further by using a simplified version of our marital status variable, as shown in Fig. 2. The estimates shown in the left and right panel are from the same model, where the common reference category is women in monogamous relationships who have not been widowed. Detailed results are shown in Table S4 of the online appendix. The results shown in Fig. 2 confirm a trend whereby the death of some members of the marriage group is worse than no deaths and the death of all members of the marriage group is clearly the worst. However, across Figs. 1 and 2, our analyses of women do not show that it is qualitatively worse to experience the death of a husband than the death of a sister wife.

#### The Role of Child Deaths and Number of Children Ever Born

The results from our full regression model, shown in Table S3 (online appendix), indicate that the hazard of mortality for women increases monotonically with increasing child deaths. Compared with women who did not experience the death of a child, the relative risk of mortality was 8% higher for women who experienced the death of one child, 14% higher for those who experienced the deaths of two children, and 20% higher for those who experienced the death of three or more children. These estimates of the main additive effects of child deaths suggest that such deaths are associated with a greater increase in mortality than the death of a husband or a sister wife. Some of these deaths will be attributable to maternal mortality.

**Fig. 3:**
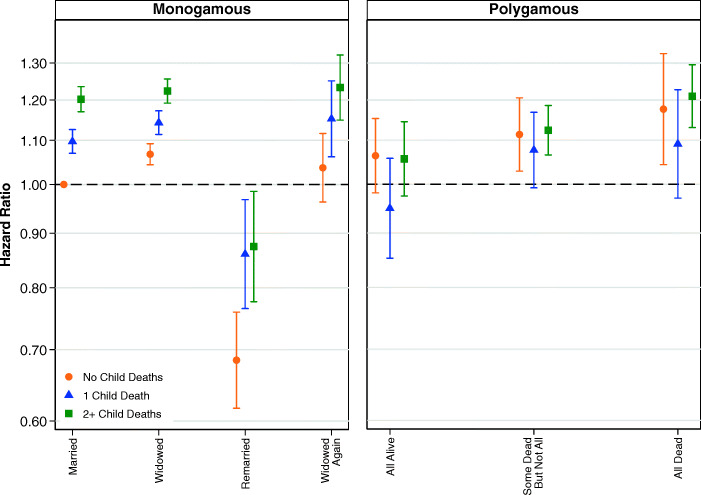
Multivariate estimates of mortality hazard for women: Interaction between child deaths and marital status of women, adjusting for parity. Error bars are 95% confidence intervals. Note that the estimates shown in the left and right panels are from the same model, and the common reference category is women in first monogamous marriages who have not been widowed. Estimates are based on Model 3.

Figure 3 shows the results from models examining the interaction between child deaths and the simplified variable for marital status. Full results are shown in Table S5 of the online appendix. Figure 3 shows that child deaths did affect the mortality of women in monogamous marriages but not the mortality of women in polygamous marriages. In monogamous marriages, it is clear that women who experienced one child death, or two or more child deaths, had higher relative risks of mortality regardless of whether they were in a first marriage, widowed, or remarried. Comparatively, in polygamous marriages, it is not at all clear that child deaths are associated with the survival of women. We are cautious about drawing firm conclusions from these results, but the larger social networks of polygamous families may provide social support that helps to moderate the negative impacts of child deaths.

**Fig. 4:**
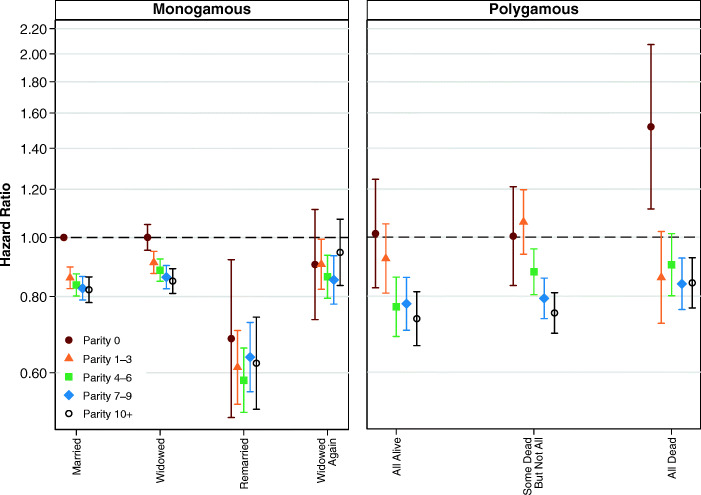
Multivariate estimates of mortality hazard for women: Interaction between parity and marital status of women, adjusting for child deaths. Error bars are 95% confidence intervals. The estimates shown in the left and right panels are from the same model, and the common reference category is women in first monogamous marriages who have not been widowed. Estimates are based on Model 4.

Although child deaths may not moderate the relationship between marital status and mortality for women in polygamous marriages, family size (indicated by number of children ever born) may be important. Figure 4 shows the results from models examining the interaction between number of children ever born and the simplified variable for marital status. A full table of results is shown in Table S6, online appendix. Net of the experience of child deaths, Fig. 4 shows that the effects of marital status do vary by a time-varying covariate for the number of children ever born. In general, childless women have higher mortality, although it is not possible for us to determine whether this can be attributed to health problems associated with infertility, or whether having more children in the household has a protective effect on health.

#### Farming Households

We also examine whether the effects of marital status on mortality are moderated by whether the husband was a farmer, and these results are available in Fig. S1 of the online appendix. Full results are shown in Table S7 in the online appendix. The results in Fig. S1 show that for women, the relative risks of mortality by marital status do not meaningfully differ by whether the husband was a farmer.

#### Marriage Order

**Fig. 5:**
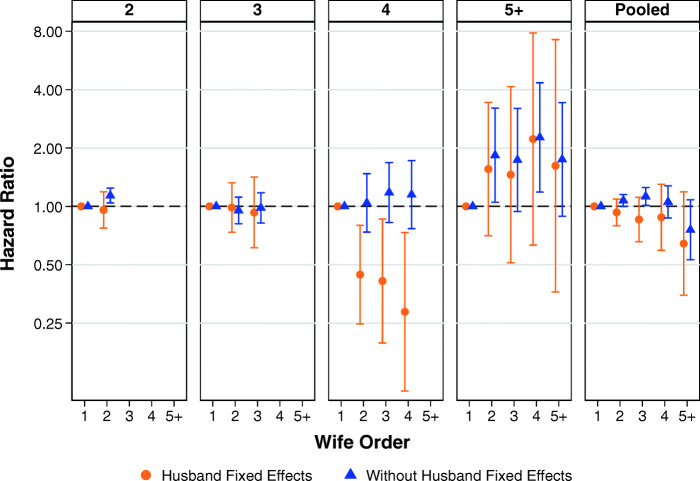
Marriage order of women in polygamous marriages and mortality by marriage group size. Error bars are 95% confidence intervals. Each panel represents a separate analysis by completed marriage group size. Estimates are based on Models 6 and 7.

We also run models to examine whether marriage order in polygamous marriages affects the mortality of women net of the mortality of the husband. We restrict these analyses to women in plural marriages. Figure 5 shows the results from these analyses in which we apply a husband fixed effect, with first wives as the reference category. In Fig. 5, each panel represents a separate analysis: that is, the models are run separately by completed marriage group size. Detailed tables of results including covariates are shown in Tables S8 and S9 in the online appendix. The results from the husband fixed-effects models for marriages with two or three sister wives—the vast majority of polygamous marriages—show that marriage order did not matter for female mortality. Higher-order wives did not have significantly different mortality from first wives, and the point estimates for second- and third-order wives are very similar to the reference category. Surprisingly, we see that higher-order wives in marriages with four sister wives have much lower mortality, but the point estimates for higher-order wives in marriages with five or more sister wives show that they have higher mortality. Given the inconsistencies in these patterns, our overall conclusion is there is no main effect of wife order on female mortality in polygamous marriages.

We also conduct additional analyses in which we interact wife order with a time-varying covariate for husband death to examine whether marriage order effects might differ before and after the death of the husband. The results from these analyses are available in Fig. S2 and Table S10 in the online appendix. These analyses are also based on husband fixed-effects models. The results from these analyses shows no statistically or substantively meaningful differences in how wife order seems to have been related to the mortality of the index person either before or after the death of the husband.

### Multivariate Regressions: Men

#### Marital Status

**Fig. 6:**
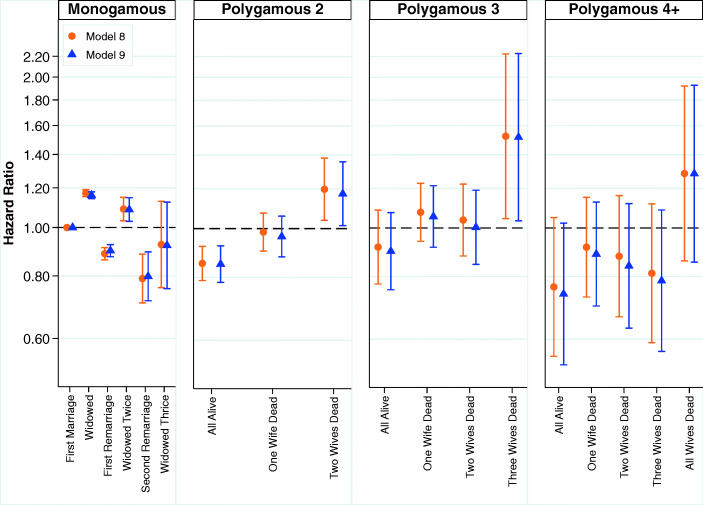
Marital status of men and mortality. Error bars are 95% confidence intervals. The estimates shown across the four panels are from the same model, and the common reference category is men in first monogamous marriages who have not been widowed. Estimates are based on Models 8 and 9.

The results from multivariate analyses examining the relationship between marital status and mortality are shown for men in Fig. 6. See Table S11, in the online appendix, for a full table of results. The estimates shown across the four panels are from the same model, and the common reference category is men in monogamous relationships who have not been widowed. The results from our analyses show that among men in monogamous marriages, being widowed increases the hazard of mortality, and remarriage serves as a marker of both positive selection and the effects of a social support resource recovered through remarriage. We observe a sawtooth pattern: men who remarry once or twice following widowhood have monotonically lower mortality both before and after being widowed. Presumably, men who are widowed but are able to remarry are healthier and may have higher status in a respect that is not captured by our adjustment for occupational status.

Looking across to the panels of Fig. 6 showing the results for men in polygamous marriages, we see that men in polygamous marriages who have not suffered the deaths of any wives have lower mortality than men in first monogamous marriages that have not been widowed. Among men in polygamous marriages who have two wives, losing one wife increases the hazard of mortality, and losing a second wife increases it further still; there appears to be a dose-response pattern in which the death of additional wives is incrementally worse. Among men with three wives, having one or two wives die increases the hazard of mortality relative to having no wives die, but losing all three wives most dramatically increases the hazard of mortality. Among men with four wives, the measurement is much less precise because of the relatively small number of these marriage groups, but the death of all four wives clearly and substantially increases the hazard of mortality for the husband substantially. Indeed, based on the point estimates, it is worse for men in polygamous marriages to lose all their wives to mortality than it is for men in monogamous marriages to lose their only wife. As with our analyses of women, we find that the estimates are very similar across Models 1 and 2, indicating that our variables for SES and fertility do not play an important confounding or mediating role in the relationship between marital status and mortality in historical Utah.

**Fig. 7:**
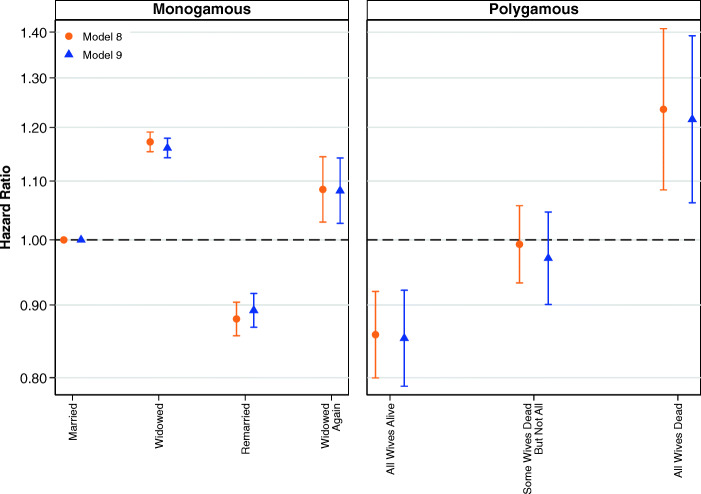
Multivariate estimates of mortality hazard for men using simplified marital status variable. Error bars are 95% confidence intervals. The estimates shown in the left and right panels are from the same model, and the common reference category is men in first monogamous marriages who have not been widowed. Estimates are based on Models 8 and 9.

Figure 7 emphasizes this pattern in our analyses using a simplified version of marital status, collapsing polygamous marital states into three categories: (1) all wives are alive; (2) some of but not all the wives died; and (3) all wives have died. Here, it is very clear that mortality is lowest in polygamous marriages when all the wives are alive and highest when they have all died. Full results are shown in Table S12 in the online appendix.

#### The Role of Child Deaths and Number of Children Ever Born

**Fig. 8:**
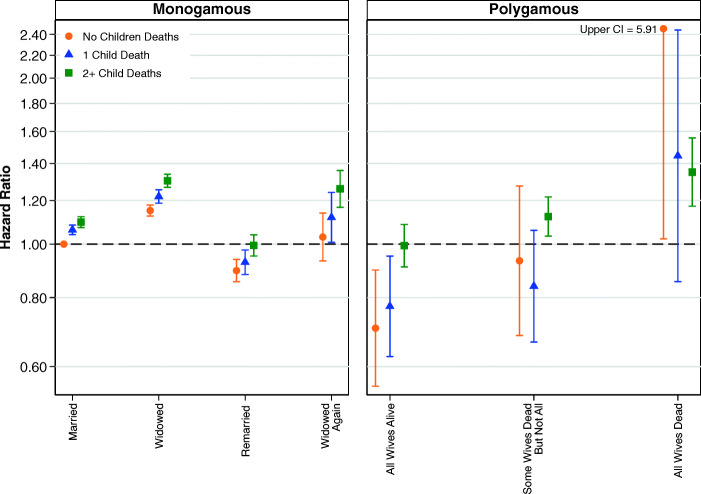
Multivariate estimates of mortality hazard for men: Interaction between child deaths and marital status of men, adjusting for parity. Error bars are 95% confidence intervals. The estimates shown in the left and right panels are from the same model, and the common reference category is men in first monogamous marriages who have not been widowed. Estimates are based on Model 10.

We now turn to analyses examining whether child deaths moderate the association between marital status and mortality for men. The results from our full regression model, shown in Table S11 in the online appendix, indicate that the hazard of mortality for men increases with child deaths. Compared with men who did not experience the death of a child, the relative risk of mortality is 6% higher for men who experienced the death of one child, 10% higher for those who experienced the deaths of two children, and 24% higher for those who experienced the deaths of five or more children. Figure 8 shows that mortality is higher among men in monogamous marriages who have experienced child deaths than among men who have not experienced child deaths. However, for men in polygamous marriages, the pattern is less clear. Although the point estimates show that men in polygamous marriages where none of their wives have died have higher mortality if more children have died, the point estimates do not follow the same direction in polygamous households where men have experienced the deaths of at least some of their wives. However, the relative infrequency of combinations of polygamy and child deaths means that no firm conclusions can be drawn from these results. Full results are shown in Table S13, online appendix.

**Fig. 9:**
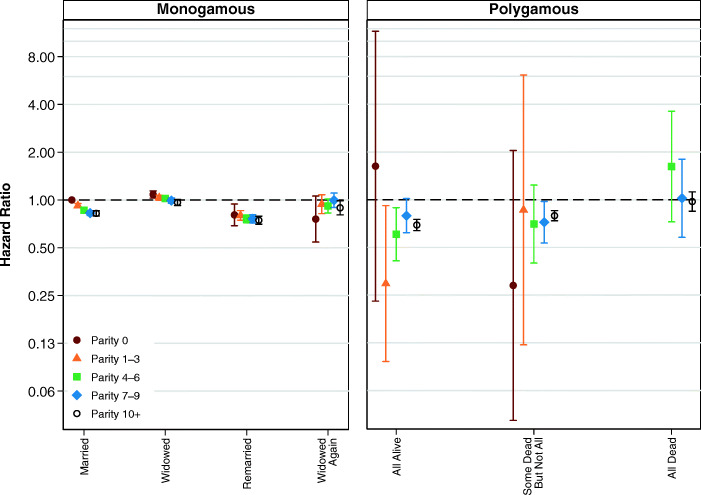
Multivariate estimates of mortality hazard for men: Interaction between parity and marital status of men, adjusting for child deaths. Error bars are 95% confidence intervals. The estimates shown in the left and right panels are from the same model, and the common reference category is women in first monogamous marriages who have not been widowed. Estimates are based on Model 11.

The results from analyses examining how number of children ever born interacts with marital status for men are shown in Fig. 9, with the full table of results available in Table S14 in the online appendix. These estimates suggest that men in monogamous marriages who are childless or who have one to three children may have slightly higher mortality. However, in most cases, these differences are not statistically significant, and it is also not possible to know whether such patterns are attributable to health problems associated with infertility or whether the presence of children has some kind of protective effect on health. For men in polygamous relationships, this association is even more difficult to disentangle because so few men with multiple wives have less than four children. However, we find no clear statistically significant or substantively meaningful differences in the hazard of mortality among men who have had 4 to 6 children, 7 to 9 children, or more than 10 children.

#### Farming Households

We also examine whether the effects of marital status on mortality are moderated by whether the man was a farmer, and these results are shown in Fig. S3 in the online appendix. Full results are shown in Table S15 in the online appendix. The results in Fig. S3 show that men who were farmers have significantly lower mortality when they have not experienced any bereavement in both monogamous and polygamous marriages; however, after experiencing any bereavement, they have hazards of mortality that are very similar to those of men who were not farmers. This may be indicative of the challenge of maintaining farm productivity in the event of the loss of adult family members.

## Discussion

The results from this study corroborate previous research demonstrating the widowhood mortality effect in historical Utah in monogamous marriages (Mineau et al. 2002) while extending that research by examining polygamous marriages. We find that women in polygamous marriages have lower mortality than women in monogamous marriages, consistent with contemporary research suggesting that polygamy does not negatively affect the health of women (Lawson et al. 2015). Our results suggest that the death of a husband or sister wife increases mortality for women, but these results do not suggest that it is qualitatively worse for women in polygamous marriages to experience the death of a husband than the death of a sister wife. Women would often have been likely to develop close bonds with their sister wives as well as their husband (Young 1970: chapter 8), meaning that the loss of either would induce a similar bereavement experience. Sister wives also likely would have provided each other with social and emotional support in the event of the husband dying. Although the resources in a polygamous marriage would have to be divided among a larger number of individuals following the death of the husband, the collective social networks of the sister wives may have been successful in drawing in sufficient resources to prevent dramatically poorer living conditions for the surviving members of the polygamous marriage.

Women who experience the deaths of their husband and all their sister wives have similar mortality to women in monogamous marriages who experience widowhood. This suggests that it may be the loss of emotional and social support that underlies the widowhood effect on mortality in this context. Furthermore, the death of the husband as well as all sister wives is likely to reflect a confluence of negative conditions that substantially increases the hazard of mortality for the surviving woman, including shared environmental factors that increase the mortality of all members of the marriage group, as well as the loss of financial resources, social and emotional support, and extended social networks that had come from the deceased husband and sister wives. Our analyses of marriage order in polygamous marriages show no statistically significant differences in mortality by order for wives, although the point estimates suggested that third- and higher-order wives may have had lower mortality.

Regarding the results for men, we find that men in polygamous marriages had lower mortality than men in monogamous marriages. Although we do adjust for the occupational status of the man, it is possible that our measure fails to fully capture the advantage associated with the greater resources available to men who were able to take multiple wives. This is further emphasized by our finding that men in polygamous marriages who have been widowed once have a very similar hazard of mortality to men in monogamous marriages who have not been widowed at all. Relative to men in their respective marriage types who had not experienced spousal death, the overall negative effect of widowhood for men in monogamous and polygamous marriages was similar. However, after we examine the effect of losing one wife versus two or more, it seems that the death of one wife for a man in a polygamous marriage was associated with a lower increase in the hazard of mortality than the death of a wife for a man in a monogamous marriage.

The fact that losing one wife was less consequential for men in polygamous marriages than in monogamous marriages makes sense from the perspective that the surviving man in a polygamous marriage would still have surviving wives who would be able to offer social and psychological support. Most men in our analytical population in polygamous marriages had two wives. As a result, the death of two wives would mean an equivalent loss of spousal support to the death of one wife for a man in a monogamous marriage, and we find that the hazard of mortality for polygamous men with two wives following the death of both wives was approximately equivalent to the hazard of mortality for men in monogamous marriages whose only wife died. The general dose-response pattern that we observe in the analyses of men in polygamous marriages suggests that both confounding and a genuine casual effect may drive our results for men. The increase in the hazard of mortality for men following the death of only some wives may reflect the contribution of shared hazardous environmental conditions, as well as assortative mating on factors that may increase the hazard of mortality. The death of all wives may reflect even worse shared environmental conditions but is also consistent with a genuine causal effect of bereavement. Indeed, losing multiple deeply valued family members may well have a more severe negative effect on the surviving husband than losing one.

To our surprise, child deaths were associated with higher mortality for men and women in married or widowed in monogamous relationships, but not for men and women in polygamous marriages with or without the experience of husband or sister wife bereavement. However, our analyses show that having a larger number of children ever born was associated with lower mortality among women who had been widowed in both monogamous and polygamous marriages. Although previous research using historical data has found that the death of a husband was particularly detrimental for women when they had multiple children (Alter et al. 2007), our findings are more consistent with previous research that shows that the presence of kin, including children, can be supportive in the event of partner loss (Mineau et al. 2002; O’Bryant 1988), although we cannot disentangle low levels of childbearing from health problems potentially associated with infertility. Indeed, a central finding of our study is that the presence of other kin in the household—a second wife, a sister wife, or children—seems to mitigate the negative effects of bereavement.

In general, our results are highly consistent with previous research on this topic: we observe that the point estimates for the increase in the hazard of mortality associated with spousal death were greater for men than for women (Shor et al. 2012). This is also consistent with the broader literature showing that men experience greater health benefits from marriage than do women, which may be driven by the tendency for women to form stronger social support networks outside the wife-husband dyad (Stevens 1995). Our results are also consistent with previous research showing that the widowhood mortality effect was often weaker in historical contexts than it has been observed to be in contemporary contexts (Mineau et al. 2002; Shor et al. 2012). In a number of senses, this is surprising given that nineteenth-century Utah would have been a relatively less-forgiving environment in which to lose critical social and economic support. However, there may have been greater support from the religious and local community, or from kin, in these historical settings. One may also consider that when mortality rates are higher overall, as in historical societies, a stronger effect would be required to increase those rates by the same factor as that observed as in contemporary lower-mortality settings.

Although this study focuses on polygamous relationships, a clear and consistent finding has been that individuals in monogamous marriages who were widowed but remarried had lower mortality than those in a first monogamous marriage, a result that was also observed in a previous study using the UPDB (Mineau et al. 2002). This pattern highlights the potentially important role of selection processes driving the association between marital status and mortality more generally. Men and women who were able to find a new marriage partner after the death of the first spouse are likely to have been characterized by some combination of physical attractiveness, ample material resources, good health, and youth. This could explain why they would have lower mortality than those who were widowed and did not find a new marriage partner but could also explain why they would even have lower mortality than those in a first marriage.

A more surprising result is our finding of no significant differences in the hazards of mortality by marriage order. Previous research has suggested a hierarchy of influence and status among women in polygamous marriages that has a tangible influence on the health of the wife as well as her children (Bove and Valeggia 2009; Gibson and Mace 2007). Future research might explore this dynamic further by examining how both fertility and the mortality of children vary by the marriage order of the mother in polygamous marriages both before and after widowhood. Because children—particularly infants—are likely to be more vulnerable to resource scarcity, this might provide some insights into how resources are distributed by wife marriage order within the household before and after the death of the husband.

Although our study focuses on marriages in nineteenth-century Utah, polygamous unions continue to be *de jure* or *de facto* tolerated in the twenty-first century across much of Africa and the Middle East, an area populated by more than a billion inhabitants. Although research has addressed many dimensions of polygamous unions in contemporary settings, including the consequences for children and intimate partner violence, the effect of widowhood on the surviving partners in contemporary polygamous unions has not been addressed to date. The health and mortality rates of widowed individuals is an important issue, but data limitations mean that examining the extent to which the patterns that we observe hold true in contemporary polygamous unions is a challenge. Our research on polygamous unions in nineteenth-century Utah using a high-quality data source suggests that the mortality effects of widowhood on the surviving partner(s) differ between monogamous and polygamous unions and indeed suggests that the effects of the loss of a single marriage partner in polygamous unions is less severe than it is in monogamous unions because of the moderating effect of surviving marriage partners.

## Electronic supplementary material

ESM 1 (PDF 188 kb)

## Data Availability

UPDB data contributed to this project. Special attention is given to protect individuals and their information contained within the UPDB and the organizations that contribute data while also allowing access to researchers. Accordingly, the Utah Resource for Genetic and Epidemiologic Research (RGE), established in 1982 by Executive Order of the Governor of Utah, administers access to the UPDB through a review process of all proposals using UPDB data. The protection of privacy and confidentiality of individuals represented in these records has been negotiated with agreements between RGE and data contributors. Data from the UPDB is available only for approved health-related research studies; access is project-specific and granted after review and approval by an RGE oversight committee and the University of Utah Institutional Review Board. This process allows researchers with approved protocols to use the data, a process that has proven effective and successful as evidenced by hundreds of approved studies that have relied on the UPDB. Requests for UPDB data used in this study will be reviewed by the RGE.

## Footnotes

1 The Utah Territory acquired statehood in 1896.

2 In a polygynous marriage, each wife is a “sister wife” to the other woman.
