# Supplementary material for: The Effects of Marital Status, Fertility, and Bereavement on Adult Mortality in Polygamous and Monogamous Households: Evidence From the Utah Population Database
Source: Demography. 2020 Sep 15;57(6):2169–98. doi: 10.1007/s13524-020-00918-z (PMC7732802; doi:10.1007/s13524-020-00918-z)
Supplement: Supplementary file 1 — (PDF 188 kb) [file 13524_2020_918_MOESM1_ESM.pdf]

Online Appendix

The Effects of Marital Status, Fertility, and Bereavement on Adult Mortality in Polygamous and Monogamous Households: Evidence From the Utah Population Database

Kieron J. Barclay, Robyn Donrovich Thorén, Heidi A. Hanson, and Ken R. Smith

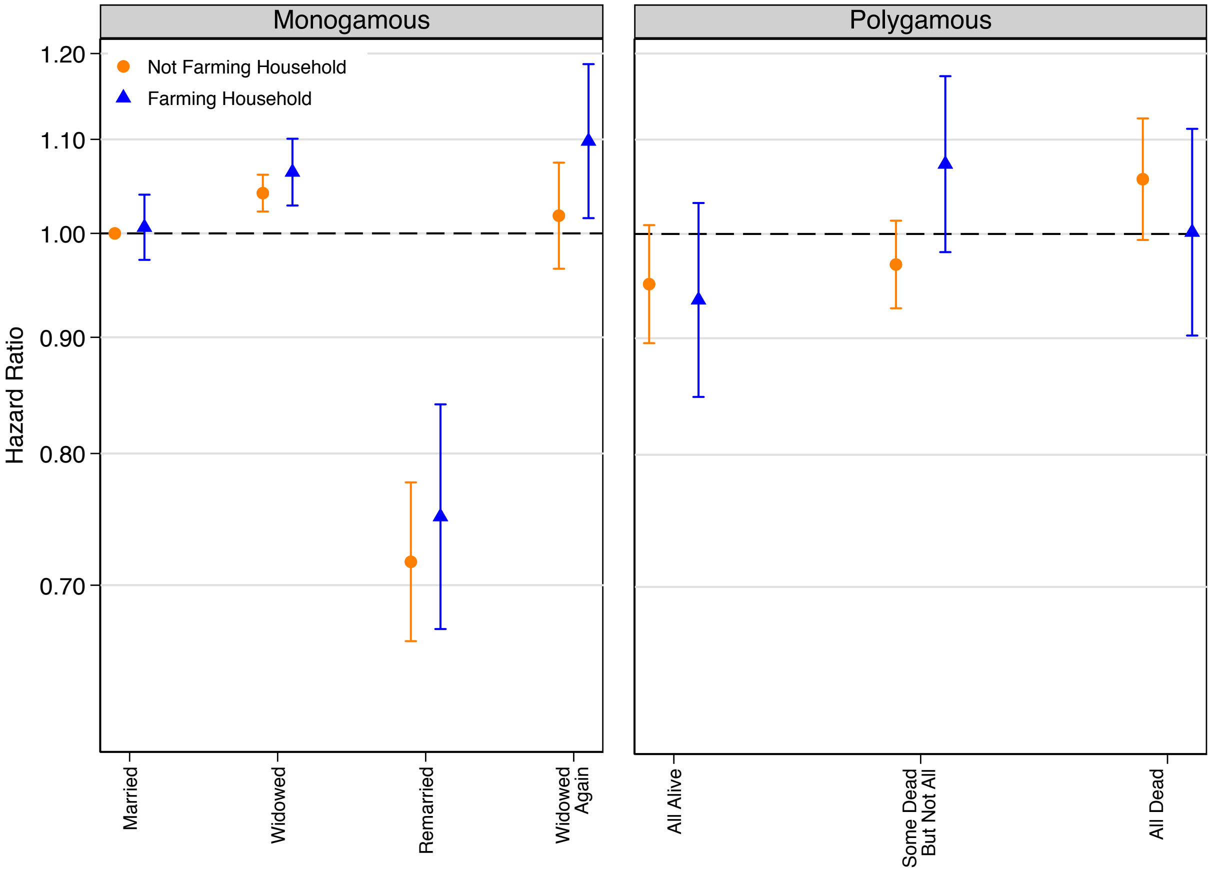

FIGURE S1. Multivariate estimates of mortality hazard for women: interaction between whether husband was a farmer and marital status of women. Error bars are 95% confidence intervals. Note that the estimates shown in the left and right panel are from the same model, where the common reference category is women in first monogamous marriages who have not been widowed. Estimates are based upon Model 4.

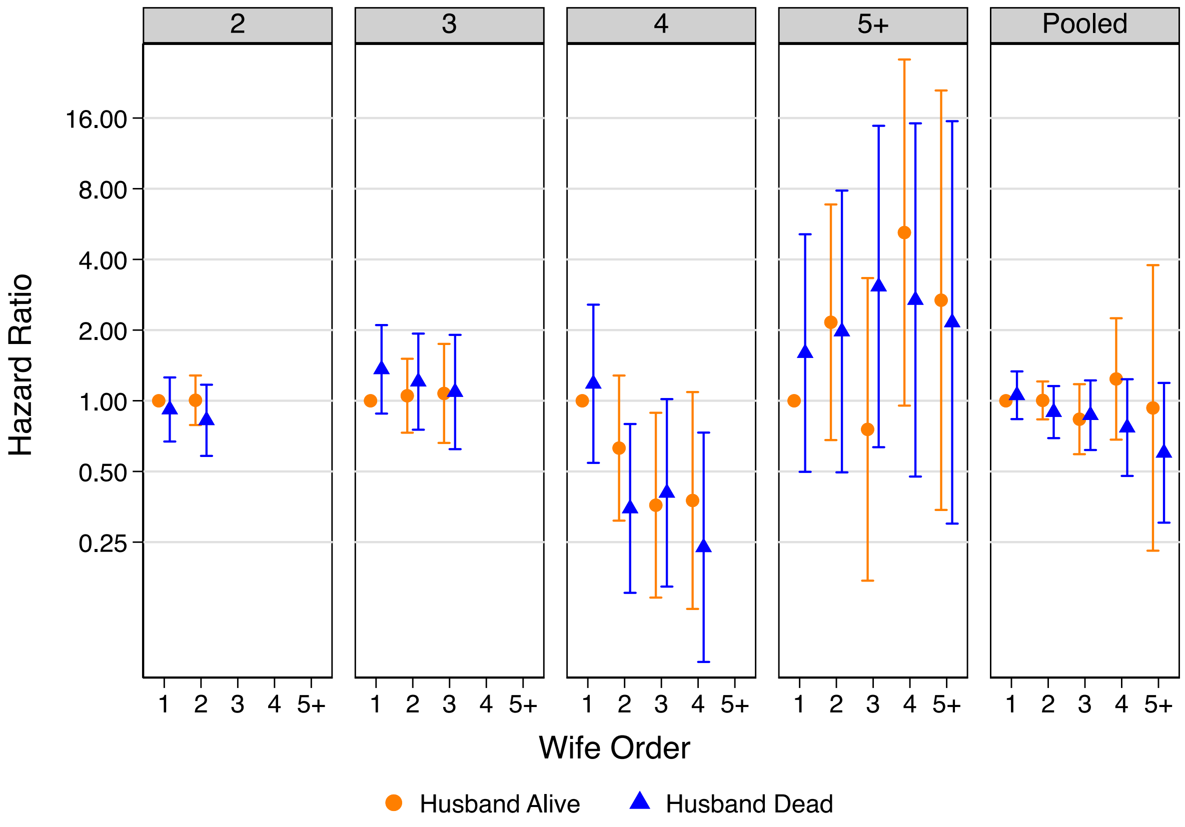

FIGURE S2. Interaction between marriage order of women in polygamous marriages and husband mortality, by marriage group size, applying husband fixed effects. Error bars are 95% confidence intervals. Each panel represents a separate analysis by completed marriage group size.

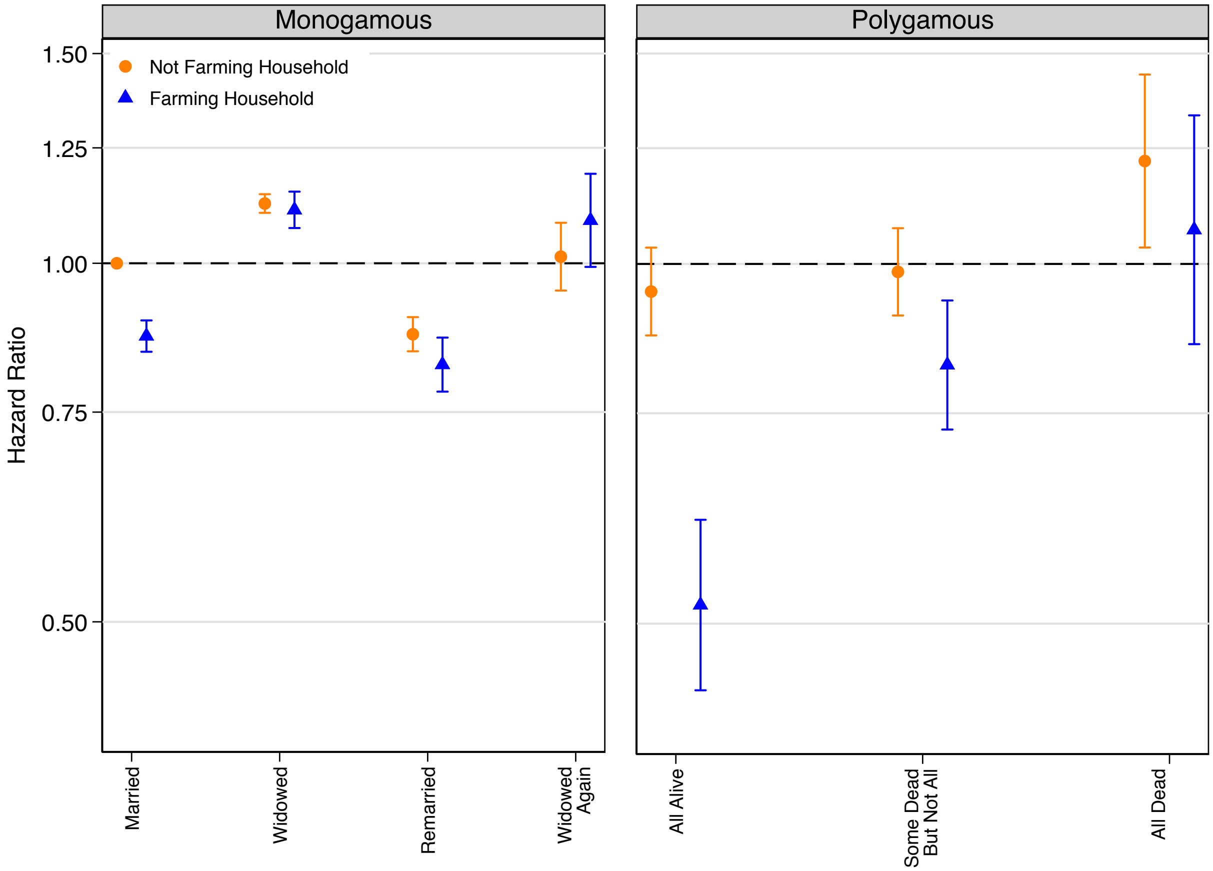

FIGURE S3. Multivariate estimates of mortality hazard for men: interaction between whether man was a farmer and marital status of men. Error bars are 95% confidence intervals. Note that the estimates shown in the left and right panel are from the same model, where the common reference category is men in first monogamous marriages who have not been widowed. Estimates are based on Model 12.

TABLE S1. Descriptive Statistics for Analytical Population: Women

		Monogamous			Polygamous		
		Person-years (%)	Deaths	Rate (10^{-2})	Person-years (%)	Deaths	Rate (10^{-2})
Marital Status	Monogamous, first marriage	68.56	39,674	1.02			
	Monogamous, first widowhood	19.55	58,743	5.30			
	Monogamous, remarried	1.36	931	1.21			
	Monogamous, widowed again	0.64	2,252	6.24			
	Polygamous, 2 wives, husband and sister wife alive				1.80	1,011	0.99
	Polygamous, 2 wives, husband alive, sister wife dead				0.22	251	2.02
	Polygamous, 2 wives, husband dead, sister wife alive				0.35	750	3.80
	Polygamous, 2 wives, husband dead, sister wife dead				0.27	1,166	7.60
	Polygamous, 3 wives, husband and sister wives alive				0.73	392	0.95
	Polygamous, 3 wives, husband alive, one sister wife dead				0.17	138	1.39
	Polygamous, 3 wives, husband alive, two sister wives dead				0.02	28	3.08
	Polygamous, 3 wives, husband dead, sister wives alive				0.17	318	3.37
	Polygamous, 3 wives, husband dead, one sister wife dead				0.17	546	5.73
	Polygamous, 3 wives, husband dead, two sister wives dead				0.07	304	8.08
	Polygamous, 4+ wives, husband and sister wives alive				0.42	219	0.92
	Polygamous, 4+ wives, husband alive, some sister wives dead				0.21	184	1.55
	Polygamous, 4+ wives, husband alive, all sister wives dead				0.00	4	4.96
	Polygamous, 4+ wives, husband dead, all sister wives alive				0.09	128	2.40
	Polygamous, 4+ wives, husband dead, some sister wives dead				0.24	701	5.08
	Polygamous, 4+ wives, husband dead, all sister wives dead				0.02	104	12.05
Birth cohort	<1800	0.54	659	2.16	0.01	19	4.92
	1800-1809	0.75	887	2.10	0.03	74	3.78
	1810-1819	1.28	1,553	2.14	0.18	305	2.97
	1820-1829	1.97	2,386	2.14	0.50	700	2.49
	1830-1839	2.78	3,297	2.09	1.10	1,364	2.19
	1840-1849	4.07	4,718	2.05	1.15	1,449	2.22
	1850-1859	7.24	8,395	2.05	1.10	1,306	2.10
	1860-1869	11.17	12,966	2.05	0.67	788	2.07
	1870-1879	15.21	17,617	2.04	0.13	162	2.23
	1880-1889	19.78	22,293	1.99	0.07	69	1.87
	1890-1900	25.34	26,829	1.87	0.01	8	1.54
Church affiliation	Not affiliated	14.56	14,333	1.74	0.39	489	2.21
	Inactive	19.16	13,582	1.25	0.27	269	1.78
	Active	56.40	73,685	2.31	4.28	5,486	2.26
Age difference between wife and husband	< -9	12.89	14,938	2.05	2.72	3,164	2.06
	-9 to -5	26.28	29,174	1.96	1.06	1,368	2.28
	-4 to -1	28.34	31,280	1.95	0.64	898	2.49
	-1 to 1	17.49	19,755	1.99	0.36	529	2.62
	1 to 4	3.80	4,644	2.16	0.11	167	2.78
	5 to 9	1.09	1,470	2.38	0.05	89	2.99
	> 9	0.18	272	2.74	0.01	29	3.59
	Missing	0.06	67	2.14			0.00
Biological parity	0	8.68	7,954	1.62	0.27	253	1.64
	1	8.82	7,064	1.41	0.23	168	1.28
	2	9.66	8,346	1.52	0.28	223	1.42
	3	9.96	9,405	1.67	0.31	286	1.60
	4	9.69	10,139	1.85	0.35	348	1.74
	5	8.74	9,777	1.98	0.42	466	1.94
	6	7.78	9,441	2.14	0.45	540	2.12
	7	6.65	8,646	2.29	0.47	594	2.25
	8	5.64	7,792	2.44	0.47	658	2.47
	9	4.74	6,992	2.61	0.47	691	2.57
	10+	9.76	16,044	2.90	1.21	2,017	2.95
Child deaths	0	53.46	44,114	1.46	1.95	1,581	1.43
	1	19.88	25,324	2.25	1.15	1,266	1.94
	2	9.17	15,032	2.90	0.81	1,204	2.61
	3	4.17	8,383	3.55	0.48	876	3.22
	4	1.91	4,482	4.13	0.28	609	3.90
	5+	1.53	4,265	4.92	0.27	708	4.68
Born outside of Utah	No	56.45	62,841	1.97	1.12	1,311	2.07
	Yes	33.66	38,759	2.03	3.83	4,933	2.28
Husband Nam-Powers score	1	0.53	594	1.98	0.00	5	2.92
	2	1.43	1,629	2.01	0.05	63	2.13
	3	4.88	5,552	2.01	0.08	94	2.13
	4	2.17	2,458	2.00	0.07	86	2.19
	5	28.53	32,272	2.00	1.27	1,592	2.21
	6	4.69	5,205	1.96	0.08	105	2.28
	7	3.78	4,101	1.92	0.14	177	2.22
	8	5.58	6,046	1.91	0.10	121	2.17
	9	3.10	3,336	1.90	0.06	69	2.10
	10	2.16	2,313	1.89	0.07	88	2.33
	Missing	33.26	38,092	2.02	3.03	3,844	2.24
Husband farmer	No	66.72	75,076	1.99	3.83	4,845	2.23
	Yes	23.39	26,522	2.00	1.12	1,399	2.21
Age at first marriage	10-19	31.93	34,535	1.91	2.59	3,094	2.11
	20-24	41.91	46,503	1.96	1.59	2,030	2.25
	25-29	11.59	13,829	2.11	0.49	679	2.46
	30-34	2.92	3,834	2.32	0.18	262	2.54
	35-39	1.00	1,446	2.55	0.06	104	2.94
	40-44	0.37	587	2.82	0.02	39	3.47
	45-49	0.19	352	3.28	0.01	18	4.61
	50-54	0.08	180	3.76	0.00	10	4.09
	55-59	0.05	132	4.25	0.00	4	3.57
	60+	0.06	202	5.75	0.00	4	17.45

TABLE S2. Descriptive Statistics for Analytical Population: Men

		Monogamous			Polygamous		
		Person-years (%)	Deaths	Rate (10^{-2})	Person-years (%)	Deaths	Rate (10^{-2})
Marital status	Monogamous, first marriage	85.32	66,394	1.67			
	Monogamous, first widowhood	8.21	27,219	7.14			
	Monogamous, second marriage	4.14	5,531	2.88			
	Monogamous, second widowhood	0.34	1,399	8.94			
	Monogamous, third marriage	0.18	308	3.72			
	Monogamous, third widowhood	0.02	98	9.50			
	Polygamous, two wives, both alive				0.98	656	1.44
	Polygamous, two wives, one dead				0.25	530	4.47
	Polygamous, two wives, both dead				0.04	193	10.91
	Polygamous, three wives, all alive				0.17	135	1.70
	Polygamous, three wives, one dead				0.15	222	3.28
	Polygamous, three wives, two dead				0.05	143	5.93
	Polygamous, three wives, all dead				0.00	27	13.79
	Polygamous, four plus wives, all alive				0.05	38	1.55
	Polygamous, four plus wives, one dead				0.06	74	2.83
	Polygamous, four plus wives, two dead				0.03	50	3.93
	Polygamous, four plus wives, three dead				0.01	38	6.97
	Polygamous, four plus wives, all dead				0.00	24	20.64
Birth cohort	<1800	0.59	639	2.34	0.00	8	6.12
	1800-1809	0.68	744	2.34	0.04	67	3.36
	1810-1819	1.23	1,287	2.25	0.15	212	3.01
	1820-1829	2.02	2,112	2.25	0.35	424	2.60
	1830-1839	3.08	3,101	2.17	0.45	538	2.56
	1840-1849	4.66	4,805	2.22	0.39	450	2.47
	1850-1859	8.69	8,884	2.20	0.30	318	2.28
	1860-1869	12.86	13,405	2.24	0.08	86	2.46
	1870-1879	16.85	17,847	2.28	0.02	24	2.22
	1880-1889	21.94	22,742	2.23	0.00	3	2.06
	1890-1900	25.59	25,383	2.13	0.00		0.00
Church affiliation	Not affiliated	19.20	18,523	2.08	0.05	51	2.26
	Inactive	20.04	16,478	1.77	0.04	38	1.92
	Active	58.96	65,948	2.41	1.70	2,041	2.58
Biological parity	0	10.30	8,116	1.70	0.00	2	0.92
	1	10.47	7,577	1.56	0.01	1	0.28
	2	11.12	8,754	1.69	0.02	1	0.14
	3	11.23	9,845	1.89	0.02	2	0.19
	4	10.55	10,115	2.06	0.03	11	0.69
	5	9.35	9,713	2.24	0.05	19	0.88
	6	8.05	9,014	2.41	0.05	14	0.62
	7	6.81	8,135	2.57	0.06	28	0.97
	8	5.62	7,215	2.76	0.07	35	1.12
	9	4.60	6,361	2.98	0.08	56	1.50
	10	3.53	5,209	3.17	0.10	83	1.86
	11-14	5.98	9,686	3.49	0.40	410	2.18
	15-19	0.56	1,106	4.22	0.50	692	2.96
	20+	0.04	103	5.03	0.40	776	4.16
Child deaths	0	60.77	48,600	1.72	0.28	111	0.84
	1	20.94	24,851	2.55	0.30	175	1.25
	2	9.17	13,370	3.14	0.32	297	2.02
	3	4.03	7,036	3.76	0.26	327	2.75
	4	1.88	3,689	4.22	0.20	282	3.07
	5+	1.42	3,403	5.17	0.44	938	4.60
Born outside of Utah	No	61.67	63,043	2.20	0.26	283	2.31
	Yes	36.54	37,906	2.23	1.53	1,847	2.60
Nam-Powers occupational score	1	0.59	675	2.46	0.00	2	4.16
	2	1.67	1,638	2.12	0.02	25	2.18
	3	5.17	5,876	2.45	0.03	34	2.25
	4	2.56	2,577	2.17	0.03	30	2.20
	5	32.16	31,702	2.12	0.60	603	2.16
	6	5.26	5,677	2.32	0.04	39	2.24
	7	4.33	4,331	2.15	0.06	65	2.28
	8	6.52	6,418	2.12	0.04	42	2.11
	9	3.67	3,521	2.07	0.02	24	2.19
	10	2.37	2,341	2.13	0.03	29	2.40
	Missing	33.92	36,193	2.30	0.92	1,237	2.91
Farmer	No	72.06	75,351	2.25	1.26	1,595	2.72
	Yes	26.15	25,598	2.11	0.53	535	2.17
Age at first marriage	10-19	5.15	4,832	2.02	0.19	216	2.51
	20-24	48.18	46,540	2.08	1.10	1,258	2.47
	25-29	30.71	31,724	2.22	0.38	472	2.65
	30-34	9.24	10,566	2.46	0.10	131	2.95
	35-39	2.90	3,806	2.82	0.02	28	3.29
	40-44	1.10	1,645	3.22	0.01	10	3.21
	45-49	0.48	803	3.62	0.01	11	4.02
	50-54	0.24	471	4.14	0.00	2	5.16
	55-59	0.11	263	5.02	0.00	1	8.37
	60+	0.09	299	7.47	0.00	1	79.06

TABLE S3. Multivariate estimates from Models 1 and 2 of mortality hazard for women using detailed marital status variable.

		Model 1			Model 2		
		HR	SE	95% CI	HR	SE	95% CI
Marital status	Monogamous, first marriage (ref)	1.00			1.00		
	Monogamous, first widowhood	1.06	0.01	1.04-1.08	1.05	0.01	1.03-1.06
	Monogamous, remarried	0.73	0.02	0.68-0.78	0.73	0.02	0.68-0.78
	Monogamous, widowed again	1.06	0.02	1.01-1.11	1.04	0.02	1.00-1.09
	Polygamous, 2 wives, husband and sister wife alive	0.95	0.03	0.89-1.01	0.95	0.03	0.89-1.01
	Polygamous, 2 wives, husband alive, sister wife dead	1.02	0.07	0.90-1.16	1.01	0.06	0.89-1.15
	Polygamous, 2 wives, husband dead, sister wife alive	0.98	0.04	0.91-1.05	0.96	0.04	0.89-1.03
	Polygamous, 2 wives, husband dead, sister wife dead	1.06	0.03	1.00-1.13	1.04	0.03	0.98-1.11
	Polygamous, 3 wives, husband and sister wives alive	0.94	0.05	0.85-1.04	0.94	0.05	0.85-1.04
	Polygamous, 3 wives, husband alive, one sister wife dead	0.88	0.08	0.75-1.04	0.87	0.07	0.74-1.03
	Polygamous, 3 wives, husband alive, two sister wives dead	1.16	0.22	0.80-1.68	1.15	0.22	0.79-1.66
	Polygamous, 3 wives, husband dead, sister wives alive	1.03	0.06	0.92-1.15	1.01	0.06	0.90-1.13
	Polygamous, 3 wives, husband dead, one sister wife dead	1.12	0.05	1.02-1.22	1.09	0.05	1.00-1.18
	Polygamous, 3 wives, husband dead, two sister wives dead	1.06	0.06	0.94-1.19	1.03	0.06	0.92-1.15
	Polygamous, 4+ wives, husband and sister wives alive	0.95	0.06	0.83-1.08	0.94	0.06	0.83-1.08
	Polygamous, 4+ wives, husband alive, some sister wives dead	1.01	0.08	0.87-1.17	1.01	0.07	0.87-1.16
	Polygamous, 4+ wives, husband alive, all sister wives dead	0.84	0.42	0.31-2.23	0.85	0.42	0.32-2.26
	Polygamous, 4+ wives, husband dead, all sister wives alive	0.98	0.09	0.82-1.16	0.96	0.09	0.81-1.14
	Polygamous, 4+ wives, husband dead, some sister wives dead	0.98	0.04	0.91-1.06	0.95	0.04	0.88-1.03
	Polygamous, 4+ wives, husband dead, all sister wives dead	1.13	0.11	0.93-1.37	1.11	0.11	0.91-1.35
Birth cohort	<1800	1.33	0.05	1.23-1.44	1.33	0.05	1.23-1.43
	1800-1809	1.25	0.04	1.17-1.33	1.23	0.04	1.15-1.32
	1810-1819	1.43	0.03	1.37-1.50	1.40	0.04	1.33-1.47
	1820-1829	1.39	0.03	1.33-1.44	1.36	0.03	1.30-1.41
	1830-1839	1.32	0.02	1.28-1.37	1.30	0.02	1.25-1.34
	1840-1849	1.29	0.02	1.26-1.33	1.27	0.02	1.23-1.31
	1850-1859	1.24	0.02	1.21-1.27	1.22	0.02	1.19-1.25
	1860-1869	1.20	0.01	1.17-1.22	1.17	0.01	1.15-1.20
	1870-1879	1.14	0.01	1.11-1.16	1.12	0.01	1.10-1.15
	1880-1889 (ref)	1.00			1.00		
	1890-1900	0.81	0.01	0.79-0.82	0.82	0.01	0.80-0.83
Church affiliation	No affiliation (ref)	1.00			1.00		
	Inactive	0.90	0.01	0.88-0.93	0.89	0.01	0.87-0.91
	Active	0.93	0.01	0.92-0.95	0.93	0.01	0.91-0.95
Age difference between wife and husband	< -9				1.01	0.01	0.99-1.03
	-9 to -5				1.00	0.01	0.98-1.02
	-4 to -1				1.00	0.01	0.98-1.02
	-1 to 1 (ref)				1.00		
	1 to 4				1.02	0.02	0.99-1.05
	5 to 9				1.01	0.03	0.96-1.07
	> 9				0.96	0.06	0.85-1.08
	Missing				1.06	0.13	0.83-1.35
Biological parity	0 (ref)				1.00		
	1				0.91	0.02	0.88-0.94
	2				0.89	0.01	0.86-0.92
	3				0.87	0.01	0.84-0.89
	4				0.88	0.01	0.85-0.90
	5				0.85	0.01	0.83-0.88
	6				0.84	0.01	0.81-0.87
	7				0.85	0.01	0.82-0.88
	8				0.83	0.01	0.80-0.86
	9				0.83	0.02	0.80-0.86
	10+				0.82	0.01	0.80-0.85
Child deaths	0 (ref)				1.00		
	1				1.08	0.01	1.06-1.10
	2				1.14	0.01	1.11-1.16
	3				1.18	0.02	1.15-1.21
	4				1.21	0.02	1.18-1.25
	5+				1.22	0.02	1.18-1.26
Born outside of Utah	No (ref)				1.00		
	Yes				0.97	0.01	0.96-0.98
Husband Nam-Powers score	1				1.05	0.04	0.96-1.14
	2				1.01	0.03	0.96-1.07
	3				1.06	0.02	1.02-1.10
	4				1.03	0.02	0.98-1.08
	5 (ref)				1.00		
	6				1.02	0.02	0.98-1.06
	7				0.94	0.02	0.90-0.98
	8				0.94	0.02	0.91-0.98
	9				0.90	0.02	0.87-0.94
	10				0.86	0.02	0.82-0.90
	Missing				1.04	0.01	1.01-1.07
Husband farmer	No (ref)				1.00		
	Yes				1.02	0.01	0.99-1.05
N			110,890			110,890	
Deaths			107,842			107,842	

TABLE S4. Multivariate estimates from Models 1 and 2 of mortality hazard for women using simplified marital status variable.

		Model 1			Model 2		
		HR	SE	95% CI	HR	SE	95% CI
Marital status	Monogamous, first marriage (ref)	1.00			1.00		
	Monogamous, first widowhood	1.06	0.01	1.04 - 1.08	1.05	0.01	1.03 - 1.06
	Monogamous, remarried	0.73	0.02	0.68 - 0.78	0.73	0.02	0.68 - 0.78
	Monogamous, widowed again	1.06	0.02	1.01 - 1.11	1.04	0.02	1.00 - 1.09
	Polygamous, husband and all sister wives alive	0.95	0.02	0.90 - 1.00	0.95	0.02	0.90 - 0.99
	Polygamous, some household members dead	1.01	0.02	0.97 - 1.05	0.99	0.02	0.95 - 1.03
	Polygamous, husband and all sister wives dead	1.06	0.03	1.01 - 1.12	1.04	0.03	0.99 - 1.10
Birth cohort	<1800	1.33	0.05	1.23 - 1.44	1.33	0.05	1.23 - 1.43
	1800-1809	1.25	0.04	1.17 - 1.33	1.23	0.04	1.15 - 1.31
	1810-1819	1.43	0.03	1.37 - 1.50	1.40	0.04	1.33 - 1.47
	1820-1829	1.39	0.03	1.33 - 1.44	1.36	0.03	1.30 - 1.41
	1830-1839	1.32	0.02	1.28 - 1.37	1.30	0.02	1.25 - 1.34
	1840-1849	1.29	0.02	1.26 - 1.33	1.27	0.02	1.23 - 1.31
	1850-1859	1.24	0.02	1.21 - 1.27	1.22	0.02	1.19 - 1.25
	1860-1869	1.20	0.01	1.17 - 1.22	1.18	0.01	1.15 - 1.20
	1870-1879	1.14	0.01	1.11 - 1.16	1.12	0.01	1.10 - 1.15
	1880-1889 (ref)	1.00			1.00		
	1890-1900	0.81	0.01	0.79 - 0.82	0.82	0.01	0.80 - 0.83
Church affiliation	No affiliation (ref)	1.00			1.00		
	Inactive	0.90	0.01	0.88 - 0.93	0.89	0.01	0.87 - 0.91
	Active	0.93	0.01	0.92 - 0.95	0.93	0.01	0.91 - 0.95
Age difference between wife and husband	< -9				1.01	0.01	0.99 - 1.03
	-9 to -5				1.00	0.01	0.98 - 1.02
	-4 to -1				1.00	0.01	0.98 - 1.02
	-1 to 1 (ref)				1.00		
	1 to 4				1.02	0.02	0.99 - 1.05
	5 to 9				1.01	0.03	0.96 - 1.07
	> 9				0.96	0.06	0.85 - 1.08
	Missing				1.06	0.13	0.83 - 1.35
Biological parity	0 (ref)				1.00		
	1				0.91	0.02	0.88 - 0.94
	2				0.89	0.01	0.86 - 0.92
	3				0.87	0.01	0.84 - 0.89
	4				0.88	0.01	0.85 - 0.90
	5				0.85	0.01	0.83 - 0.88
	6				0.84	0.01	0.81 - 0.87
	7				0.85	0.01	0.82 - 0.88
	8				0.83	0.01	0.80 - 0.86
	9				0.83	0.02	0.80 - 0.86
	10+				0.82	0.01	0.80 - 0.85
Child deaths	0 (ref)				1.00		
	1				1.08	0.01	1.06 - 1.10
	2				1.14	0.01	1.11 - 1.16
	3				1.18	0.02	1.15 - 1.21
	4				1.21	0.02	1.18 - 1.25
	5+				1.22	0.02	1.18 - 1.26
Born outside of Utah	No (ref)				1.00		
	Yes				0.97	0.01	0.96 - 0.98
Husband Nam-Powers score	1				1.05	0.04	0.96 - 1.14
	2				1.01	0.03	0.96 - 1.07
	3				1.06	0.02	1.02 - 1.10
	4				1.03	0.02	0.98 - 1.08
	5 (ref)				1.00		
	6				1.02	0.02	0.98 - 1.06
	7				0.94	0.02	0.90 - 0.98
	8				0.94	0.02	0.91 - 0.98
	9				0.90	0.02	0.87 - 0.94
	10				0.86	0.02	0.82 - 0.90
	Missing				1.04	0.01	1.01 - 1.07
Husband farmer	No (ref)				1.00		
	Yes				1.02	0.01	0.99 - 1.05
N		110,890			110,890		
Deaths		107,842			107,842		

Table S5: Multivariate estimates from Model 3 of mortality hazard for women using interaction between simplified marital status variable and number of child deaths.

		Model 3		
		HR	SE	95% CI
Child deaths x Marital status	0 Deaths & Monogamous, first marriage (ref)	1.00		
	0 Deaths & Monogamous, first widowhood	1.07	0.01	1.04 - 1.09
	0 Deaths & Monogamous, remarried	0.68	0.04	0.62 - 0.76
	0 Deaths & Monogamous, widowed again	1.04	0.04	0.96 - 1.12
	0 Deaths & Polygamous, husband and all sister wives alive	1.06	0.04	0.98 - 1.15
	0 Deaths & Polygamous, some household members dead	1.11	0.05	1.03 - 1.21
	0 Deaths & Polygamous, husband and all sister wives dead	1.18	0.07	1.04 - 1.33
	1 Death & Monogamous, first marriage	1.10	0.01	1.07 - 1.13
	1 Death & Monogamous, first widowhood	1.14	0.01	1.11 - 1.17
	1 Death & Monogamous, remarried	0.86	0.05	0.76 - 0.97
	1 Death & Monogamous, widowed again	1.15	0.05	1.06 - 1.25
	1 Death & Polygamous, husband and all sister wives alive	0.95	0.05	0.85 - 1.06
	1 Death & Polygamous, some household members dead	1.08	0.04	0.99 - 1.17
	1 Death & Polygamous, husband and all sister wives dead	1.09	0.07	0.97 - 1.23
	2+ Deaths & Monogamous, first marriage	1.20	0.02	1.17 - 1.24
	2+ Deaths & Monogamous, first widowhood	1.22	0.02	1.19 - 1.26
	2+ Deaths & Monogamous, remarried	0.87	0.05	0.78 - 0.99
	2+ Deaths & Monogamous, widowed again	1.23	0.04	1.15 - 1.32
	2+ Deaths & Polygamous, husband and all sister wives alive	1.06	0.04	0.97 - 1.14
	2+ Deaths & Polygamous, some household members dead	1.12	0.03	1.07 - 1.19
	2+ Deaths & Polygamous, husband and all sister wives dead	1.21	0.04	1.13 - 1.29
Birth cohort	<1800	1.33	0.05	1.23 - 1.44
	1800-1809	1.23	0.04	1.15 - 1.32
	1810-1819	1.40	0.04	1.34 - 1.48
	1820-1829	1.36	0.03	1.30 - 1.42
	1830-1839	1.30	0.02	1.26 - 1.35
	1840-1849	1.28	0.02	1.24 - 1.32
	1850-1859	1.22	0.02	1.19 - 1.25
	1860-1869	1.18	0.01	1.15 - 1.20
	1870-1879	1.12	0.01	1.10 - 1.15
	1880-1889 (ref)	1.00		
	1890-1900	0.82	0.01	0.80 - 0.83
Church affiliation	No affiliation (ref)	1.00		
	Inactive	0.89	0.01	0.87 - 0.91
	Active	0.93	0.01	0.91 - 0.95
Age difference between wife and husband	< -9	1.01	0.01	0.99 - 1.03
	-9 to -5	1.00	0.01	0.98 - 1.02
	-4 to -1	1.00	0.01	0.98 - 1.02
	-1 to 1 (ref)	1.00		
	1 to 4	1.02	0.02	0.99 - 1.05
	5 to 9	1.02	0.03	0.96 - 1.07
	> 9	0.96	0.06	0.85 - 1.08
	Missing	1.06	0.13	0.84 - 1.35
Biological parity	0 (ref)	1.00		
	1	0.91	0.02	0.88 - 0.94

Continued on next page

Table S5 – *Continued from previous page*

		Model 3		
		HR	SE	95% CI
	2	0.89	0.01	0.86 - 0.92
	3	0.87	0.01	0.84 - 0.89
	4	0.88	0.01	0.85 - 0.90
	5	0.85	0.01	0.82 - 0.88
	6	0.84	0.01	0.81 - 0.87
	7	0.85	0.01	0.82 - 0.88
	8	0.84	0.02	0.81 - 0.87
	9	0.83	0.02	0.80 - 0.86
	10+	0.84	0.01	0.81 - 0.86
Born outside of Utah	No (ref)	1.00		
	Yes	0.97	0.01	0.96 - 0.98
Husband Nam-Powers score	1	1.05	0.05	0.96 - 1.14
	2	1.01	0.03	0.96 - 1.07
	3	1.06	0.02	1.02 - 1.10
	4	1.03	0.02	0.98 - 1.08
	5 (ref)	1.00		
	6	1.02	0.02	0.98 - 1.06
	7	0.94	0.02	0.90 - 0.98
	8	0.94	0.02	0.91 - 0.98
	9	0.90	0.02	0.87 - 0.94
	10	0.86	0.02	0.82 - 0.90
	Missing	1.04	0.01	1.01 - 1.07
Husband farmer	No (ref)	1.00		
	Yes	1.02	0.01	0.99 - 1.05
N			110,890	
Deaths			107,842	

Table S6: Multivariate estimates from Model 4 of mortality hazard for women using interaction between simplified marital status variable and number of children ever born.

		Model 4		
		HR	SE	95% CI
Parity x Marital status	Parity 0 & Monogamous, first marriage (ref)	1.00		
	Parity 0 & Monogamous, first widowhood	1.00	0.02	0.95-1.05
	Parity 0 & Monogamous, remarried	0.68	0.10	0.51-0.92
	Parity 0 & Monogamous, widowed again	0.90	0.10	0.73-1.11
	Parity 0 & Polygamous, husband and all sister wives alive	1.01	0.11	0.83-1.24
	Parity 0 & Polygamous, some household members dead	1.00	0.10	0.83-1.21
	Parity 0 & Polygamous, husband and all sister wives dead	1.52	0.24	1.11-2.07
	Parity 1-3 & Monogamous, first marriage	0.86	0.02	0.82-0.89
	Parity 1-3 & Monogamous, first widowhood	0.91	0.02	0.87-0.95
	Parity 1-3 & Monogamous, remarried	0.61	0.04	0.53-0.70
	Parity 1-3 & Monogamous, widowed again	0.90	0.04	0.82-0.99
	Parity 1-3 & Polygamous, husband and all sister wives alive	0.92	0.06	0.81-1.05
	Parity 1-3 & Polygamous, some household members dead	1.06	0.07	0.94-1.20
	Parity 1-3 & Polygamous, husband and all sister wives dead	0.86	0.08	0.72-1.02
	Parity 4-6 & Monogamous, first marriage	0.84	0.02	0.80-0.87
	Parity 4-6 & Monogamous, first widowhood	0.88	0.02	0.85-0.92
	Parity 4-6 & Monogamous, remarried	0.58	0.04	0.52-0.66
	Parity 4-6 & Monogamous, widowed again	0.86	0.04	0.79-0.94
	Parity 4-6 & Polygamous, husband and all sister wives alive	0.77	0.04	0.69-0.86
	Parity 4-6 & Polygamous, some household members dead	0.88	0.04	0.80-0.96
	Parity 4-6 & Polygamous, husband and all sister wives dead	0.90	0.05	0.80-1.01
	Parity 7-9 & Monogamous, first marriage	0.83	0.02	0.79-0.86
	Parity 7-9 & Monogamous, first widowhood	0.86	0.02	0.82-0.90
	Parity 7-9 & Monogamous, remarried	0.64	0.04	0.56-0.73
	Parity 7-9 & Monogamous, widowed again	0.85	0.04	0.78-0.93
	Parity 7-9 & Polygamous, husband and all sister wives alive	0.78	0.04	0.70-0.86
	Parity 7-9 & Polygamous, some household members dead	0.79	0.03	0.73-0.86
	Parity 7-9 & Polygamous, husband and all sister wives dead	0.84	0.04	0.76-0.92
	Parity 10+ & Monogamous, first marriage	0.82	0.02	0.78-0.86
	Parity 10+ & Monogamous, first widowhood	0.85	0.02	0.81-0.89
	Parity 10+ & Monogamous, remarried	0.62	0.06	0.52-0.74
	Parity 10+ & Monogamous, widowed again	0.95	0.06	0.83-1.07
	Parity 10+ & Polygamous, husband and all sister wives alive	0.73	0.04	0.66-0.81
	Parity 10+ & Polygamous, some household members dead	0.75	0.03	0.70-0.81
	Parity 10+ & Polygamous, husband and all sister wives dead	0.84	0.04	0.76-0.92
Birth cohort	<1800	1.32	0.05	1.22-1.43
	1800-1809	1.22	0.04	1.15-1.31
	1810-1819	1.39	0.04	1.32-1.46
	1820-1829	1.35	0.03	1.29-1.41
	1830-1839	1.29	0.02	1.25-1.34
	1840-1849	1.27	0.02	1.23-1.31
	1850-1859	1.21	0.02	1.18-1.24
	1860-1869	1.17	0.01	1.15-1.20
	1870-1879	1.12	0.01	1.10-1.14
	1880-1889 (ref)	1.00		

Continued on next page

Table S6 – *Continued from previous page*

		Model 4		
		HR	SE	95% CI
	1890-1900	0.82	0.01	0.80-0.83
Church affiliation	No affiliation (ref)	1.00		
	Inactive	0.89	0.01	0.87-0.91
	Active	0.93	0.01	0.91-0.94
Age difference between wife and husband	< -9	1.01	0.01	0.99-1.03
	-9 to -5	1.00	0.01	0.98-1.02
	-4 to -1	1.00	0.01	0.98-1.02
	-1 to 1 (ref)	1.00		
	1 to 4	1.02	0.02	0.99-1.05
	5 to 9	1.02	0.03	0.96-1.07
	> 9	0.96	0.06	0.85-1.08
	Missing	1.06	0.13	0.84-1.35
Child deaths	0 (ref)	1.00		
	1	1.07	0.01	1.06-1.09
	2	1.13	0.01	1.10-1.15
	3	1.17	0.02	1.14-1.20
	4	1.21	0.02	1.17-1.25
	5+	1.21	0.02	1.17-1.26
Born outside of Utah	No (ref)	1.00		
	Yes	0.97	0.01	0.96-0.98
Husband Nam-Powers score	1	1.04	0.04	0.96-1.14
	2	1.01	0.03	0.96-1.07
	3	1.06	0.02	1.02-1.10
	4	1.03	0.02	0.98-1.08
	5 (ref)	1.00		
	6	1.02	0.02	0.98-1.06
	7	0.94	0.02	0.90-0.98
	8	0.94	0.02	0.91-0.98
	9	0.90	0.02	0.87-0.94
	10	0.86	0.02	0.82-0.90
	Missing	1.04	0.01	1.01-1.07
Husband farmer	No (ref)	1.00		
	Yes	1.02	0.01	0.99-1.04
N		110,890		
Deaths		107,842		

Table S7: Multivariate estimates from Model 5 of mortality hazard for women using interaction between simplified marital status variable and whether husband is a farmer

		Model 5		
		HR	SE	95% CI
Husband farmer x Marital status	Not a Farmer & Monogamous, first marriage	1.00		
	Not a Farmer & Monogamous, first widowhood	1.04	0.01	1.02-1.06
	Not a Farmer & Monogamous, remarried	0.72	0.03	0.66-0.78
	Not a Farmer & Monogamous, widowed again	1.02	0.03	0.96-1.07
	Not a Farmer & Polygamous, husband and all sister wives alive	0.95	0.03	0.90-1.01
	Not a Farmer & Polygamous, some household members dead	0.97	0.02	0.93-1.01
	Not a Farmer & Polygamous, husband and all sister wives dead	1.06	0.03	0.99-1.12
	Farmer & Monogamous, first marriage	1.01	0.02	0.97-1.04
	Farmer & Monogamous, first widowhood	1.06	0.02	1.03-1.10
	Farmer & Monogamous, remarried	0.75	0.04	0.67-0.84
	Farmer & Monogamous, widowed again	1.10	0.04	1.02-1.19
	Farmer & Polygamous, husband and all sister wives alive	0.94	0.05	0.85-1.03
	Farmer & Polygamous, some household members dead	1.07	0.05	0.98-1.17
	Farmer & Polygamous, husband and all sister wives dead	1.00	0.05	0.90-1.11
Birth cohort	<1800	1.33	0.05	1.23-1.43
	1800-1809	1.23	0.04	1.15-1.31
	1810-1819	1.40	0.04	1.33-1.47
	1820-1829	1.36	0.03	1.30-1.41
	1830-1839	1.30	0.02	1.25-1.34
	1840-1849	1.27	0.02	1.23-1.31
	1850-1859	1.22	0.02	1.19-1.25
	1860-1869	1.18	0.01	1.15-1.20
	1870-1879	1.12	0.01	1.10-1.15
	1880-1889 (ref)	1.00		
	1890-1900	0.82	0.01	0.80-0.83
Church affiliation	No affiliation (ref)	1.00		
	Inactive	0.89	0.01	0.87-0.91
	Active	0.93	0.01	0.91-0.95
Age difference between wife and husband	< -9	1.01	0.01	0.99-1.03
	-9 to -5	1.00	0.01	0.98-1.02
	-4 to -1	1.00	0.01	0.98-1.02
	-1 to 1 (ref)	1.00		
	1 to 4	1.02	0.02	0.99-1.05
	5 to 9	1.02	0.03	0.96-1.07
	> 9	0.96	0.06	0.85-1.08
	Missing	1.06	0.13	0.83-1.35
Biological parity	0 (ref)	1.00		
	1	0.91	0.02	0.88-0.94
	2	0.89	0.01	0.86-0.92
	3	0.87	0.01	0.84-0.89
	4	0.88	0.01	0.85-0.90
	5	0.85	0.01	0.83-0.88
	6	0.84	0.01	0.81-0.87
	7	0.85	0.01	0.82-0.88
	8	0.83	0.01	0.80-0.86

Continued on next page

Table S7 – *Continued from previous page*

		Model 5		
		HR	SE	95% CI
	9	0.83	0.02	0.80-0.86
	10+	0.82	0.01	0.80-0.85
Child deaths	0 (ref)	1.00		
	1	1.08	0.01	1.06-1.10
	2	1.14	0.01	1.11-1.16
	3	1.18	0.02	1.15-1.21
	4	1.21	0.02	1.17-1.25
	5+	1.22	0.02	1.18-1.26
Born outside of Utah	No (ref)	1.00		
	Yes	0.97	0.01	0.96-0.98
Husband Nam-Powers score	1	1.05	0.04	0.96-1.14
	2	1.01	0.03	0.96-1.07
	3	1.06	0.02	1.02-1.10
	4	1.03	0.02	0.98-1.08
	5 (ref)	1.00		
	6	1.02	0.02	0.98-1.06
	7	0.94	0.02	0.90-0.98
	8	0.94	0.02	0.91-0.98
	9	0.90	0.02	0.87-0.94
	10	0.86	0.02	0.82-0.90
	Missing	1.04	0.01	1.01-1.07
N		110,890		
Deaths		107,842		

TABLE S8. Multivariate estimates from Model 6 of mortality hazard for women by marriage order using Cox proportional hazard model without stratifying on shared husband ID.

		Model 6														
		Marriage size = 2			Marriage size = 3			Marriage size = 4			Marriage size = 5+			Marriage size = Pooled		
		HR	SE	95% CI	HR	SE	95% CI	HR	SE	95% CI	HR	SE	95% CI	HR	SE	95% CI
Wife order	1 (ref)	1.00			1.00			1.00			1.00			1.00		
	2	1.14	0.05	1.04-1.24	0.95	0.08	0.81-1.12	1.04	0.18	0.74-1.47	1.84	0.52	1.05-3.21	1.07	0.04	1.00-1.15
	3				0.98	0.09	0.82-1.18	1.18	0.21	0.83-1.68	1.74	0.54	0.94-3.20	1.12	0.06	1.01-1.25
	4							1.15	0.24	0.77-1.72	2.27	0.75	1.18-4.34	1.05	0.10	0.87-1.28
	5										1.75	0.60	0.89-3.43	0.76	0.14	0.53-1.08
Birth year		1.00	0.00	0.99-1.00	1.00	0.00	0.99-1.00	1.00	0.01	0.99-1.01	0.98	0.01	0.97-1.00	1.00	0.00	1.00-1.00
Husband dead	No (ref)	1.00			1.00			1.00			1.00			1.00		
	Yes	1.07	0.06	0.96-1.20	1.23	0.11	1.02-1.47	1.07	0.21	0.73-1.58	1.02	0.32	0.56-1.88	1.10	0.05	1.00-1.20
Husband Nam-Powers	1	4.34	3.55	0.88-21.55										4.61	3.62	0.99-21.47
score	2	1.10	0.24	0.71-1.69	0.76	0.26	0.40-1.48	0.79	0.42	0.28-2.24				0.93	0.16	0.67-1.29
	3	0.86	0.16	0.60-1.23	0.62	0.21	0.31-1.21	0.41	0.33	0.08-2.02				0.77	0.12	0.57-1.03
	4	0.94	0.19	0.63-1.40	1.11	0.44	0.51-2.41	1.01	0.73	0.25-4.15	4.33	2.43	1.45-12.98	1.04	0.17	0.76-1.43
	5	1.00			1.00			1.00			1.00			1.00		
	6	1.00	0.18	0.70-1.44	0.97	0.28	0.54-1.71				0.93	0.69	0.21-3.98	0.98	0.15	0.74-1.32
	7	0.99	0.16	0.72-1.35	0.95	0.21	0.61-1.47	1.52	0.78	0.56-4.14				1.01	0.12	0.79-1.28
	8	0.82	0.15	0.57-1.19	0.98	0.26	0.58-1.66	2.16	1.16	0.75-6.17	1.36	0.81	0.42-4.38	0.93	0.13	0.70-1.22
	9	0.86	0.19	0.55-1.33	0.55	0.20	0.28-1.11	6.08	4.37	1.49-24.86	0.56	0.39	0.14-2.19	0.79	0.14	0.56-1.10
	10	0.65	0.14	0.42-0.99	1.77	0.59	0.92-3.41	2.98	1.61	1.03-8.60	0.80	0.47	0.25-2.52	0.76	0.12	0.55-1.03
	Missing	1.00	0.12	0.79-1.27	0.85	0.15	0.60-1.19	1.06	0.38	0.53-2.14	1.26	0.46	0.62-2.57	0.94	0.09	0.78-1.13
Husband farmer	No	1.00			1.00			1.00						1.00		
	Yes	0.97	0.12	0.77-1.23	0.88	0.16	0.62-1.26	1.00	0.38	0.47-2.11				0.94	0.09	0.78-1.14
N			2,704			1,182			378			193			4,457	
Deaths			2,668			1,160			371			187			4,386	

TABLE S9. Multivariate estimates from Model 7 of mortality hazard for women by marriage order using Cox proportional hazard model stratifying on shared husband ID.

		Model 7														
		Marriage size = 2			Marriage size = 3			Marriage size = 4			Marriage size = 5+			Marriage size = Pooled		
		HR	SE	95% CI	HR	SE	95% CI	HR	SE	95% CI	HR	SE	95% CI	HR	SE	95% CI
Wife order	1 (ref)	1.00			1.00			1.00			1.00			1.00		
	2	0.96	0.11	0.77-1.19	0.99	0.15	0.73-1.32	0.44	0.13	0.25-0.80	1.56	0.63	0.71-3.44	0.93	0.08	0.79-1.09
	3				0.93	0.20	0.61-1.41	0.41	0.15	0.20-0.86	1.46	0.78	0.51-4.14	0.86	0.12	0.66-1.11
	4							0.29	0.14	0.11-0.73	2.22	1.43	0.63-7.83	0.88	0.18	0.59-1.30
	5										1.62	1.24	0.36-7.25	0.64	0.20	0.35-1.19
Age at first marriage		1.10	0.02	1.07-1.13	1.09	0.02	1.05-1.12	1.16	0.03	1.10-1.22	1.14	0.05	1.05-1.23	1.10	0.01	1.08-1.12
Birth year		1.02	0.01	1.00-1.04	1.00	0.01	0.98-1.03	1.06	0.02	1.02-1.11	0.98	0.03	0.92-1.04	1.01	0.01	1.00-1.03
Husband dead	No (ref)	1.00			1.00			1.00			1.00			1.00		
	Yes	0.86	0.12	0.65-1.15	1.19	0.22	0.83-1.72	0.88	0.27	0.49-1.59	1.25	0.58	0.25-0.80	0.97	0.10	0.25-0.80
N			2,704			1,182			378			193			4,457	
Deaths			2,668			1,160			371			187			4,386	

TABLE S10. Women: marriage order interacted with husband survival status and mortality, stratifying on shared husband ID.

		Marriage size = 2			Marriage size = 3			Marriage size = 4			Marriage size = 5+			Marriage size = Pooled		
Husband Alive	Wife order	HR	SE	95% CI	HR	SE	95% CI	HR	SE	95% CI	HR	SE	95% CI	HR	SE	95% CI
No	1 (ref)	1.00			1.00			1.00			1.00			1.00		
	2	1.00	0.12	0.79-1.28	1.05	0.19	0.73-1.51	0.63	0.23	0.31-1.28	2.16	1.27	0.68-6.86	1.00	0.10	0.83-1.21
	3				1.07	0.27	0.66-1.75	0.36	0.17	0.15-0.89	0.76	0.57	0.17-3.33	0.83	0.15	0.59-1.18
	4							0.38	0.20	0.13-1.09	5.20	4.51	0.95-28.41	1.24	0.38	0.68-2.25
	5										2.68	2.81	0.34-20.96	0.93	0.67	0.23-3.78
Yes	1	0.92	0.15	0.67-1.26	1.36	0.30	0.88-2.10	1.18	0.47	0.54-2.57	1.60	0.95	0.50-5.12	1.06	0.13	0.84-1.33
	2	0.83	0.15	0.58-1.17	1.21	0.29	0.75-1.93	0.35	0.15	0.15-0.80	1.97	1.39	0.50-7.86	0.89	0.12	0.69-1.16
	3				1.09	0.31	0.62-1.91	0.41	0.19	0.16-1.02	3.07	2.47	0.63-14.83	0.87	0.15	0.62-1.22
	4							0.24	0.14	0.08-0.73	2.69	2.37	0.48-15.19	0.77	0.19	0.48-1.24
	5										2.16	2.17	0.30-15.51	0.60	0.21	0.30-1.19
Age at first marriage		1.10	0.02	1.07-1.13	1.09	0.02	1.06-1.12	1.16	0.03	1.10-1.23	1.15	0.05	1.06-1.25	1.10	0.01	1.08-1.12
Birth year		1.02	0.01	1.00-1.04	1.01	0.01	0.98-1.03	1.07	0.02	1.02-1.11	0.98	0.03	0.92-1.05	1.02	0.01	1.00-1.03
N			2,704			1,182			378			193			4,457	
Deaths			2,668			1,160			371			187			4,386	

TABLE S11. Multivariate estimates from Models 8 and 9 of mortality hazard for men using detailed marital status variable.

		Model 8			Model 9		
		HR	SE	95% CI	HR	SE	95% CI
Marital status	Monogamous, first marriage (ref)	1.00			1.00		
	Monogamous, first widowhood	1.17	0.01	1.15-1.19	1.16	0.01	1.14-1.18
	Monogamous, second marriage	0.89	0.01	0.86-0.91	0.90	0.01	0.87-0.92
	Monogamous, second widowhood	1.09	0.03	1.03-1.15	1.09	0.03	1.03-1.15
	Monogamous, third marriage	0.79	0.05	0.71-0.88	0.80	0.05	0.71-0.89
	Monogamous, third widowhood	0.92	0.09	0.76-1.13	0.92	0.09	0.75-1.12
	Polygamous, two wives, both alive	0.85	0.03	0.79-0.92	0.85	0.04	0.78-0.92
	Polygamous, two wives, one dead	0.98	0.04	0.90-1.07	0.96	0.05	0.88-1.06
	Polygamous, two wives, both dead	1.20	0.09	1.04-1.38	1.17	0.09	1.01-1.36
	Polygamous, three wives, all alive	0.92	0.08	0.77-1.09	0.90	0.08	0.75-1.07
	Polygamous, three wives, one dead	1.07	0.07	0.94-1.23	1.05	0.08	0.92-1.21
	Polygamous, three wives, two dead	1.04	0.09	0.88-1.22	1.00	0.09	0.85-1.19
	Polygamous, three wives, all dead	1.52	0.29	1.04-2.23	1.52	0.30	1.03-2.23
	Polygamous, four plus wives, all alive	0.76	0.12	0.55-1.05	0.74	0.12	0.53-1.02
	Polygamous, four plus wives, one dead	0.92	0.11	0.73-1.15	0.89	0.11	0.70-1.13
	Polygamous, four plus wives, two dead	0.88	0.12	0.67-1.16	0.84	0.12	0.63-1.12
	Polygamous, four plus wives, three dead	0.81	0.13	0.59-1.12	0.78	0.13	0.57-1.09
	Polygamous, four plus wives, all dead	1.28	0.26	0.86-1.92	1.28	0.27	0.85-1.93
Birth cohort	<1800	1.03	0.04	0.95-1.11	0.98	0.04	0.90-1.06
	1800-1809	1.13	0.04	1.05-1.21	1.06	0.04	0.98-1.14
	1810-1819	1.08	0.03	1.03-1.14	1.03	0.03	0.98-1.09
	1820-1829	1.06	0.02	1.02-1.11	1.07	0.02	1.02-1.12
	1830-1839	1.04	0.02	1.00-1.07	1.09	0.02	1.05-1.13
	1840-1849	1.06	0.02	1.02-1.09	1.12	0.02	1.08-1.15
	1850-1859	1.04	0.01	1.01-1.06	1.06	0.01	1.03-1.09
	1860-1869	1.02	0.01	1.00-1.04	1.03	0.01	1.00-1.05
	1870-1879	1.06	0.01	1.04-1.08	1.05	0.01	1.03-1.07
	1880-1889 (ref)	1.00			1.00		
	1890-1900	0.87	0.01	0.85-0.88	0.87	0.01	0.85-0.88
Church affiliation	No affiliation (ref)	1.00			1.00		
	Inactive	1.00	0.01	0.98-1.03	1.00	0.01	0.98-1.02
	Active	0.81	0.01	0.80-0.82	0.83	0.01	0.82-0.85
Biological parity	0 (ref)				1.00		
	1				0.95	0.02	0.92-0.98
	2				0.94	0.01	0.91-0.97
	3				0.91	0.01	0.88-0.94
	4				0.90	0.01	0.87-0.93
	5				0.88	0.01	0.85-0.90
	6				0.87	0.01	0.84-0.90
	7				0.85	0.01	0.82-0.88
	8				0.85	0.02	0.83-0.89
	9				0.85	0.02	0.82-0.89
	10				0.86	0.02	0.82-0.89
	11-14				0.83	0.02	0.80-0.86
	15-19				0.81	0.03	0.76-0.86
	20+				0.82	0.04	0.74-0.90
Child deaths (ref)	0				1.00		
	1				1.06	0.01	1.04-1.08
	2				1.10	0.01	1.07-1.12
	3				1.12	0.02	1.09-1.15
	4				1.11	0.02	1.08-1.16
	5+				1.24	0.02	1.19-1.29
Born outside of Utah	No (ref)				1.00		
	Yes				0.92	0.01	0.91-0.93
Nam-Powers occupational score	1				1.15	0.05	1.06-1.24
	2				0.83	0.02	0.79-0.88
	3				1.09	0.02	1.05-1.13
	4				0.87	0.02	0.83-0.91
	5 (ref)				0.89	0.02	0.86-0.92
	6				1.00		
	7				0.85	0.02	0.82-0.89
	8				0.81	0.01	0.78-0.84
	9				0.78	0.02	0.75-0.81
	10				0.77	0.02	0.73-0.81
	Missing				1.04	0.01	1.01-1.07
Farmer	No (ref)				1.00		
	Yes				0.91	0.01	0.88-0.93
N		106,979			106,979		
Deaths		103,079			103,079		

TABLE S12. Multivariate estimates from Models 8 and 9 of mortality hazard for men using simplified marital status variable.

		Model 8			Model 9		
		HR	SE	95% CI	HR	SE	95% CI
Marital status	Monogamous, first marriage (ref)	1.00			1.00		
	Monogamous, first widowhood	1.17	0.01	1.15-1.19	1.16	0.01	1.14-1.18
	Monogamous, remarried	0.88	0.01	0.86-0.90	0.89	0.01	0.87-0.92
	Monogamous, widowed again	1.09	0.03	1.03-1.14	1.08	0.03	1.03-1.14
	Polygamous, husband and all sister wives alive	0.86	0.03	0.80-0.92	0.85	0.03	0.79-0.92
	Polygamous, some household members dead	0.99	0.03	0.93-1.06	0.97	0.04	0.90-1.05
	Polygamous, husband and all sister wives dead	1.24	0.08	1.08-1.41	1.22	0.08	1.06-1.39
Birth cohort	$¡$1800	1.03	0.04	0.95-1.11	0.98	0.04	0.90-1.06
	1800-1809	1.13	0.04	1.05-1.21	1.06	0.04	0.98-1.14
	1810-1819	1.08	0.03	1.03-1.14	1.03	0.03	0.98-1.09
	1820-1829	1.06	0.02	1.02-1.11	1.07	0.02	1.02-1.12
	1830-1839	1.04	0.02	1.00-1.07	1.09	0.02	1.05-1.13
	1840-1849	1.06	0.02	1.02-1.09	1.12	0.02	1.08-1.16
	1850-1859	1.04	0.01	1.01-1.06	1.06	0.01	1.04-1.09
	1860-1869	1.02	0.01	1.00-1.04	1.03	0.01	1.00-1.05
	1870-1879	1.06	0.01	1.03-1.08	1.05	0.01	1.03-1.07
	1880-1889 (ref)	1.00			1.00		
	1890-1900	0.87	0.01	0.85-0.88	0.87	0.01	0.85-0.88
Church affiliation	No affiliation (ref)	1.00			1.00		
	Inactive	1.00	0.01	0.98-1.03	1.00	0.01	0.98-1.02
	Active	0.81	0.01	0.80-0.82	0.83	0.01	0.82-0.85
Biological parity	0 (ref)				1.00		
	1				0.95	0.02	0.92-0.98
	2				0.94	0.01	0.91-0.97
	3				0.91	0.01	0.88-0.94
	4				0.90	0.01	0.87-0.93
	5				0.88	0.01	0.85-0.90
	6				0.87	0.01	0.84-0.90
	7				0.85	0.01	0.82-0.88
	8				0.85	0.02	0.83-0.89
	9				0.85	0.02	0.82-0.89
	10				0.86	0.02	0.82-0.89
	11-14				0.83	0.02	0.80-0.86
	15-19				0.81	0.03	0.76-0.86
	20+				0.81	0.04	0.74-0.89
Child deaths (ref)	0				1.00		
	1				1.06	0.01	1.04-1.08
	2				1.10	0.01	1.07-1.12
	3				1.12	0.02	1.09-1.15
	4				1.11	0.02	1.08-1.16
	5+				1.24	0.02	1.19-1.29
Born outside of Utah	No (ref)				1.00		
	Yes				0.92	0.01	0.91-0.93
Nam-Powers occupational score	1				1.14	0.05	1.06-1.24
	2				0.83	0.02	0.79-0.88
	3				1.09	0.02	1.05-1.13
	4				0.87	0.02	0.83-0.91
	5 (ref)				0.89	0.02	0.86-0.92
	6				1.00		
	7				0.85	0.02	0.82-0.88
	8				0.81	0.01	0.78-0.84
	9				0.78	0.02	0.75-0.81
	10				0.77	0.02	0.73-0.81
	Missing				1.04	0.01	1.01-1.07
Farmer	No (ref)				1.00		
	Yes				0.91	0.01	0.88-0.93
N			106,979			106,979	
Deaths			103,079			103,079	

Table S13: Multivariate estimates from Model 10 of mortality hazard for men using interaction between simplified marital status variable and number of child deaths

		Model 10		
		HR	SE	95% CI
Child deaths x Marital status	0 Deaths & Monogamous, first marriage (ref)	1.00		
	0 Deaths & Monogamous, first widowhood	1.15	0.01	1.12-1.18
	0 Deaths & Monogamous, remarried	0.90	0.02	0.85-0.94
	0 Deaths & Monogamous, widowed again	1.03	0.05	0.93-1.14
	0 Deaths & Polygamous, husband and all sister wives alive	0.70	0.09	0.55-0.90
	0 Deaths & Polygamous, some household members dead	0.93	0.15	0.68-1.27
	0 Deaths & Polygamous, husband and all sister wives dead	2.46	1.10	1.02-5.91
	1 Death & Monogamous, first marriage	1.06	0.01	1.04-1.08
	1 Death & Monogamous, first widowhood	1.22	0.02	1.19-1.25
	1 Death & Monogamous, remarried	0.93	0.02	0.88-0.98
	1 Death & Monogamous, widowed again	1.12	0.06	1.01-1.24
	1 Death & Polygamous, husband and all sister wives alive	0.77	0.08	0.63-0.95
	1 Death & Polygamous, some household members dead	0.84	0.10	0.66-1.06
	1 Death & Polygamous, husband and all sister wives dead	1.45	0.39	0.86-2.44
	2+ Deaths & Monogamous, first marriage	1.10	0.01	1.07-1.12
	2+ Deaths & Monogamous, first widowhood	1.30	0.02	1.27-1.34
	2+ Deaths & Monogamous, remarried	0.99	0.02	0.95-1.04
	2+ Deaths & Monogamous, widowed again	1.26	0.05	1.17-1.36
	2+ Deaths & Polygamous, husband and all sister wives alive	0.99	0.04	0.91-1.09
	2+ Deaths & Polygamous, some household members dead	1.12	0.05	1.03-1.22
	2+ Deaths & Polygamous, husband and all sister wives dead	1.35	0.10	1.17-1.56
Birth cohort	$¡$1800	0.98	0.04	0.90-1.06
	1800-1809	1.06	0.04	0.99-1.14
	1810-1819	1.04	0.03	0.98-1.10
	1820-1829	1.08	0.02	1.03-1.13
	1830-1839	1.09	0.02	1.05-1.14
	1840-1849	1.12	0.02	1.09-1.16
	1850-1859	1.07	0.01	1.04-1.09
	1860-1869	1.03	0.01	1.01-1.05
	1870-1879	1.05	0.01	1.03-1.07
	1880-1889 (ref)	1.00		
	1890-1900	0.87	0.01	0.85-0.88
Church affiliation	No affiliation (ref)	1.00		
	Inactive	1.00	0.01	0.98-1.02
	Active	0.83	0.01	0.82-0.85
Biological parity	0 (ref)	1.00		
	1	0.95	0.02	0.92-0.98
	2	0.94	0.01	0.91-0.97
	3	0.91	0.01	0.88-0.94
	4	0.90	0.01	0.87-0.92
	5	0.87	0.01	0.85-0.90
	6	0.87	0.01	0.84-0.90
	7	0.84	0.01	0.82-0.87
	8	0.85	0.02	0.82-0.89
	9	0.86	0.02	0.82-0.89

Continued on next page

Table S13 – *Continued from previous page*

		Model 10		
		HR	SE	95% CI
	10	0.86	0.02	0.83-0.90
	11-14	0.85	0.02	0.82-0.88
	15-19	0.83	0.03	0.78-0.88
	20+	0.85	0.04	0.77-0.93
Born outside of Utah	No (ref)	1.00		
	Yes	0.92	0.01	0.91-0.94
Nam-Powers occupational score	1	1.14	0.05	1.06-1.24
	2	0.83	0.02	0.79-0.88
	3	1.09	0.02	1.05-1.13
	4	0.87	0.02	0.83-0.91
	5 (ref)	0.89	0.02	0.85-0.92
	6	1.00		
	7	0.85	0.02	0.82-0.89
	8	0.81	0.01	0.78-0.84
	9	0.78	0.02	0.75-0.81
	10	0.77	0.02	0.73-0.81
	Missing	1.04	0.01	1.01-1.07
Farmer	No (ref)	1.00		
	Yes	0.91	0.01	0.88-0.93
N			106,979	
Deaths			103,079	

Table S14: Multivariate estimates from Model 11 of mortality hazard for men using interaction between simplified marital status variable and number of children ever born

		Model 11		
		HR	SE	95% CI
Parity x Marital status	Parity 0 & Monogamous, first marriage (ref)	1.00		
	Parity 0 & Monogamous, first widowhood	1.08	0.03	1.03-1.14
	Parity 0 & Monogamous, remarried	0.81	0.06	0.69-0.94
	Parity 0 & Monogamous, widowed again	0.76	0.13	0.54-1.06
	Parity 0 & Polygamous, husband and all sister wives alive	1.63	1.63	0.23-11.56
	Parity 0 & Polygamous, some household members dead	0.29	0.29	0.04-2.04
	Parity 0 & Polygamous, husband and all sister wives dead			
	Parity 1-3 & Monogamous, first marriage	0.92	0.01	0.89-0.95
	Parity 1-3 & Monogamous, first widowhood	1.03	0.02	1.00-1.07
	Parity 1-3 & Monogamous, remarried	0.80	0.03	0.75-0.86
	Parity 1-3 & Monogamous, widowed again	0.94	0.07	0.82-1.08
	Parity 1-3 & Polygamous, husband and all sister wives alive	0.30	0.17	0.10-0.92
	Parity 1-3 & Polygamous, some household members dead	0.86	0.86	0.12-6.12
	Parity 1-3 & Polygamous, husband and all sister wives dead			
	Parity 4-6 & Monogamous, first marriage	0.86	0.01	0.84-0.89
	Parity 4-6 & Monogamous, first widowhood	1.02	0.02	0.98-1.06
	Parity 4-6 & Monogamous, remarried	0.76	0.02	0.72-0.80
	Parity 4-6 & Monogamous, widowed again	0.92	0.05	0.83-1.01
	Parity 4-6 & Polygamous, husband and all sister wives alive	0.61	0.12	0.41-0.89
	Parity 4-6 & Polygamous, some household members dead	0.70	0.20	0.40-1.24
	Parity 4-6 & Polygamous, husband and all sister wives dead	1.62	0.66	0.72-3.61
	Parity 7-9 & Monogamous, first marriage	0.83	0.01	0.80-0.85
	Parity 7-9 & Monogamous, first widowhood	0.99	0.02	0.95-1.03
	Parity 7-9 & Monogamous, remarried	0.76	0.02	0.72-0.81
	Parity 7-9 & Monogamous, widowed again	0.99	0.05	0.90-1.10
	Parity 7-9 & Polygamous, husband and all sister wives alive	0.79	0.10	0.62-1.02
	Parity 7-9 & Polygamous, some household members dead	0.72	0.11	0.53-0.97
	Parity 7-9 & Polygamous, husband and all sister wives dead	1.02	0.30	0.58-1.80
	Parity 10+ & Monogamous, first marriage	0.82	0.02	0.79-0.85
	Parity 10+ & Monogamous, first widowhood	0.96	0.02	0.92-1.01
	Parity 10+ & Monogamous, remarried	0.75	0.02	0.70-0.79
	Parity 10+ & Monogamous, widowed again	0.89	0.05	0.81-0.99
	Parity 10+ & Polygamous, husband and all sister wives alive	0.69	0.03	0.64-0.75
	Parity 10+ & Polygamous, some household members dead	0.79	0.03	0.74-0.85
	Parity 10+ & Polygamous, husband and all sister wives dead	0.97	0.07	0.85-1.12
Birth cohort	<1800	0.98	0.04	0.90-1.06
	1800-1809	1.05	0.04	0.98-1.13
	1810-1819	1.03	0.03	0.97-1.09
	1820-1829	1.07	0.02	1.02-1.12
	1830-1839	1.09	0.02	1.05-1.13
	1840-1849	1.12	0.02	1.08-1.15
	1850-1859	1.06	0.01	1.03-1.09
	1860-1869	1.03	0.01	1.00-1.05
	1870-1879	1.05	0.01	1.03-1.07
	1880-1889 (ref)	1.00		

Continued on next page

Table S14 – *Continued from previous page*

		Model 11		
		HR	SE	95% CI
	1890-1900	0.87	0.01	0.85-0.88
Church affiliation	No affiliation (ref)	1.00		
	Inactive	1.00	0.01	0.98-1.02
	Active	0.83	0.01	0.82-0.85
Child deaths (ref)	0	1.00		
	1	1.06	0.01	1.04-1.07
	2	1.09	0.01	1.07-1.11
	3	1.11	0.02	1.08-1.14
	4	1.10	0.02	1.07-1.14
	5+	1.22	0.02	1.18-1.27
Born outside of Utah	No (ref)	1.00		
	Yes	0.92	0.01	0.91-0.93
Nam-Powers occupational score	1	1.14	0.05	1.06-1.24
	2	0.83	0.02	0.79-0.88
	3	1.09	0.02	1.05-1.13
	4	0.87	0.02	0.83-0.91
	5 (ref)	0.89	0.02	0.86-0.92
	6	1.00		
	7	0.85	0.02	0.82-0.89
	8	0.81	0.01	0.78-0.84
	9	0.78	0.02	0.75-0.81
	10	0.77	0.02	0.73-0.81
	Missing	1.04	0.01	1.01-1.07
Farmer	No (ref)	1.00		
	Yes	0.90	0.01	0.88-0.93
N		106,979		
Deaths		103,079		

Table S15: Multivariate estimates from Model 12 of mortality hazard for men using interaction between simplified marital status variable and farmer status

		Model 12		
		HR	SE	95% CI
Farmer x Marital status	Not a Farmer & Monogamous, first marriage	1.00		
	Not a Farmer & Monogamous, first widowhood	1.12	0.01	1.10-1.14
	Not a Farmer & Monogamous, remarried	0.87	0.01	0.84-0.90
	Not a Farmer & Monogamous, widowed again	1.01	0.03	0.95-1.08
	Not a Farmer & Polygamous, husband and all sister wives alive	0.95	0.04	0.87-1.03
	Not a Farmer & Polygamous, some household members dead	0.98	0.04	0.91-1.07
	Not a Farmer & Polygamous, husband and all sister wives dead	1.22	0.10	1.03-1.44
	Farmer & Monogamous, first marriage (ref)	0.87	0.01	0.84-0.90
	Farmer & Monogamous, first widowhood	1.11	0.02	1.07-1.15
	Farmer & Monogamous, remarried	0.82	0.02	0.78-0.87
	Farmer & Monogamous, widowed again	1.09	0.05	0.99-1.19
	Farmer & Polygamous, husband and all sister wives alive	0.52	0.04	0.44-0.61
	Farmer & Polygamous, some household members dead	0.82	0.05	0.73-0.93
	Farmer & Polygamous, husband and all sister wives dead	1.07	0.12	0.86-1.33
Birth cohort	$¡$1800	0.98	0.04	0.91-1.06
	1800-1809	1.05	0.04	0.98-1.13
	1810-1819	1.02	0.03	0.97-1.08
	1820-1829	1.07	0.02	1.02-1.12
	1830-1839	1.09	0.02	1.05-1.13
	1840-1849	1.12	0.02	1.08-1.16
	1850-1859	1.06	0.01	1.03-1.09
	1860-1869	1.03	0.01	1.01-1.05
	1870-1879	1.05	0.01	1.03-1.07
	1880-1889 (ref)	1.00		
	1890-1900	0.87	0.01	0.85-0.88
Church affiliation	No affiliation (ref)	1.00		
	Inactive	1.00	0.01	0.98-1.02
	Active	0.83	0.01	0.82-0.85
Biological parity	0 (ref)	1.00		
	1	0.95	0.02	0.92-0.98
	2	0.94	0.01	0.91-0.97
	3	0.91	0.01	0.88-0.94
	4	0.90	0.01	0.87-0.92
	5	0.88	0.01	0.85-0.90
	6	0.87	0.01	0.84-0.90
	7	0.85	0.01	0.82-0.87
	8	0.85	0.02	0.82-0.89
	9	0.85	0.02	0.82-0.89
	10	0.86	0.02	0.82-0.89
	11-14	0.83	0.02	0.80-0.86
	15-19	0.81	0.03	0.76-0.86
	20+	0.81	0.04	0.74-0.89
Child deaths (ref)	0	1.00		
	1	1.06	0.01	1.04-1.08
	2	1.09	0.01	1.07-1.12

Continued on next page

Table S15 – *Continued from previous page*

		Model 12		
		HR	SE	95% CI
	3	1.12	0.02	1.09-1.15
	4	1.11	0.02	1.07-1.15
	5+	1.24	0.02	1.19-1.28
Born outside of Utah	No (ref)	1.00		
	Yes	0.92	0.01	0.91-0.94
Nam-Powers occupational score	1	1.14	0.05	1.06-1.24
	2	0.83	0.02	0.79-0.88
	3	1.09	0.02	1.05-1.13
	4	0.87	0.02	0.83-0.91
	5 (ref)	0.89	0.02	0.86-0.92
	6	1.00		
	7	0.85	0.02	0.82-0.88
	8	0.81	0.01	0.78-0.84
	9	0.78	0.02	0.75-0.81
	10	0.77	0.02	0.73-0.81
	Missing	1.04	0.01	1.01-1.07
N		106,979		
Deaths		103,079		
